# Neurological and metabolic related pathophysiologies and treatment of comorbid diabetes with depression

**DOI:** 10.1111/cns.14497

**Published:** 2023-11-06

**Authors:** Sixin Li, Dong Yang, Xuhui Zhou, Lu Chen, Lini Liu, Ruoheng Lin, Xinyu Li, Ying Liu, Huiwen Qiu, Hui Cao, Jian Liu, Quan Cheng

**Affiliations:** ^1^ Department of Psychiatry, The School of Clinical Medicine Hunan University of Chinese Medicine Changsha Hunan China; ^2^ Department of Psychiatry Brain Hospital of Hunan Province (The Second People's Hospital of Hunan Province) Changsha Hunan China; ^3^ Department of Gastroenterology, The School of Clinical Medicine Hunan University of Chinese Medicine Changsha Hunan China; ^4^ Department of Gastroenterology Brain Hospital of Hunan Province (The Second People's Hospital of Hunan Province) Changsha Hunan China; ^5^ Department of Psychiatry, National Clinical Research Center for Mental Disorders The Second Xiangya Hospital of Central South University Changsha Hunan China; ^6^ Center for Medical Research and Innovation, The First Hospital, Hunan University of Chinese Medicine Changsha Hunan China; ^7^ Department of Neurosurgery, Xiangya Hospital Central South University Changsha Hunan China; ^8^ National Clinical Research Center for Geriatric Disorders, Xiangya Hospital Central South University Changsha Hunan China

**Keywords:** comorbidity, depression, diabetes mellitus, pathophysiology, treatment

## Abstract

**Background:**

The comorbidity between diabetes mellitus and depression was revealed, and diabetes mellitus increased the prevalence of depressive disorder, which ranked 13th in the leading causes of disability‐adjusted life‐years. Insulin resistance, which is common in diabetes mellitus, has increased the risk of depressive symptoms in both humans and animals. However, the mechanisms behind the comorbidity are multi‐factorial and complicated. There is still no causal chain to explain the comorbidity exactly. Moreover, Selective serotonin reuptake inhibitors, insulin and metformin, which are recommended for treating diabetes mellitus‐induced depression, were found to be a risk factor in some complications of diabetes.

**Aims:**

Given these problems, many researchers made remarkable efforts to analyze diabetes complicating depression from different aspects, including insulin resistance, stress and Hypothalamic–Pituitary–Adrenal axis, neurological system, oxidative stress, and inflammation. Drug therapy, such as Hydrogen Sulfide, Cannabidiol, Ascorbic Acid and Hesperidin, are conducive to alleviating diabetes mellitus and depression. Here, we reviewed the exact pathophysiology underlying the comorbidity between depressive disorder and diabetes mellitus and drug therapy.

**Methods:**

The review refers to the available literature in PubMed and Web of Science, searching critical terms related to diabetes mellitus, depression and drug therapy.

**Results:**

In this review, we found that brain structure and function, neurogenesis, brain‐derived neurotrophic factor and glucose and lipid metabolism were involved in the pathophysiology of the comorbidity. Obesity might lead to diabetes mellitus and depression through reduced adiponectin and increased leptin and resistin. In addition, drug therapy displayed in this review could expand the region of potential therapy.

**Conclusions:**

The review summarizes the mechanisms underlying the comorbidity. It also overviews drug therapy with anti‐diabetic and anti‐depressant effects.

## Graphical abstract

The pathophysiology and treatment in the comorbidity of diabetes mellitus and depression. GM, Gastrointestinal Microbiome; HPA, Hypothalamic–Pituitary–Adrenal. By Figdraw.

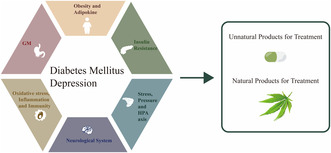

## 1 INTRODUCTION

Depression is one of the comorbidities of diabetes mellitus (DM). The prevalence of depressive disorders in patients with type 2 diabetes mellitus (T2DM) has increased from 0.70 per cent in 2000 to 1.25 per cent in 2010.^1^ T2DM has a 1.35% relative risk of depressive disorder despite symptom severity.^2^ 28.2% had increased their HbA1c since the pandemic began and 18.2% had uncontrolled T2DM.^3^ Among the top 25 causes of disability‐adjusted life years (DALYs), depressive disorders ranked 13th, according to statistics from the Global Burden of Disease (GBD) 2019.^4^ It is estimated that major depressive disorder caused 49.4 million DALYs globally due to the COVID‐19 pandemic in 2020.^5^

People experience increased levels of depressive and anxiety disorders in a highly stressful society.^6^, ^7^ Developments in neuropsychiatry have identified depression as a risk factor for some diseases, such as Alzheimer's.^8^, ^9^ Diabetes and depression have a bidirectional relationship, increasing each other's risk. Individuals with T2DM are at higher risk of experiencing depressive symptoms and displaying elevated levels of hyperglycaemic markers, as evidenced by the Maastricht Study.^10^ Conversely, it is feasible that depression could lead to the development of diabetes due to an elevated incidence of central obesity. Thus, depression appears to contribute to the progression of diabetes.^11^ Depression is a notable factor contributing to dementia and cognitive impairment in individuals with diabetes.^12^, ^13^ A cohort study has pointed out that people with diabetes and depressive disorder tend to develop hyperglycemic crisis episodes (HCE).^14^

The current understanding of depression is primarily centered around receptor theory. This includes the N‐methyl‐D‐aspartate receptor, α‐amino‐3‐hydroxy‐5‐methyl‐4‐isoxazolepropionic acid receptor, glucocorticoid receptor, 5‐hydroxytryptamine receptor, GABAA receptor α2, and dopamine receptor. Thus, the mainstream therapy for depression in DM is SSRIs, which remain 5‐HT at the receptor sites to exert an anti‐depression effect.^15^ However, the explicit mechanisms behind the comorbidity of DM and depression are more complicated than depression, as much research has revealed that emotions affected our capacity to control our body.^16^, ^17^ The recent attention towards the pathophysiology underlying DM with depression focused on insulin resistance, oxidative stress, inflammation and the nervous system.^18^ For instance, high insulin resistance in patients with T2DM had a close association with depressive symptoms.^19^ Furthermore, a higher systemic immune‐inflammation (SII) index had been found in patients with DM and depressive disorder.^20^ The numerous articles exploring other mechanisms, such as the HPA axis and adipokine, suggest the diversity of mechanisms behind comorbidity. Higher depression severity was found to correlate positively with higher midnight cortisol levels.^21^ Patients with diabetes presented the worst glycemia control, the most obese and the worst executive functions, and the reduction of executive functions resulted from depressive symptoms.^22^ Also, the relationship between uncontrolled diabetes and depression had been possibly mediated by central adiposity.^11^ The main goal of treatment in comorbidity is to improve glycemic control and alleviate depression symptoms. However, the above findings suggested that the mainstream therapy for depression, such as SSRIs,^23^ focusing on receptor therapy, found it hard to meet the multi‐faceted pathology of depression in DM. It was found that not all treatments, which are adequate to attenuate depressive symptoms, can control blood glucose levels.^24^ For example, sertraline has been proven to alleviate depression but does not affect blood glucose.^25^ Analyses about the drug with both anti‐diabetic and anti‐depressant effects increased. For instance, CBD treatment reduced depressive symptoms, as evidenced by the altered level of 5‐HT, NA and/or DA, and reduced glycemia, as evidenced by a remarkable increase in weight gain and the level of insulin.^26^, ^27^

**FIGURE 1:**
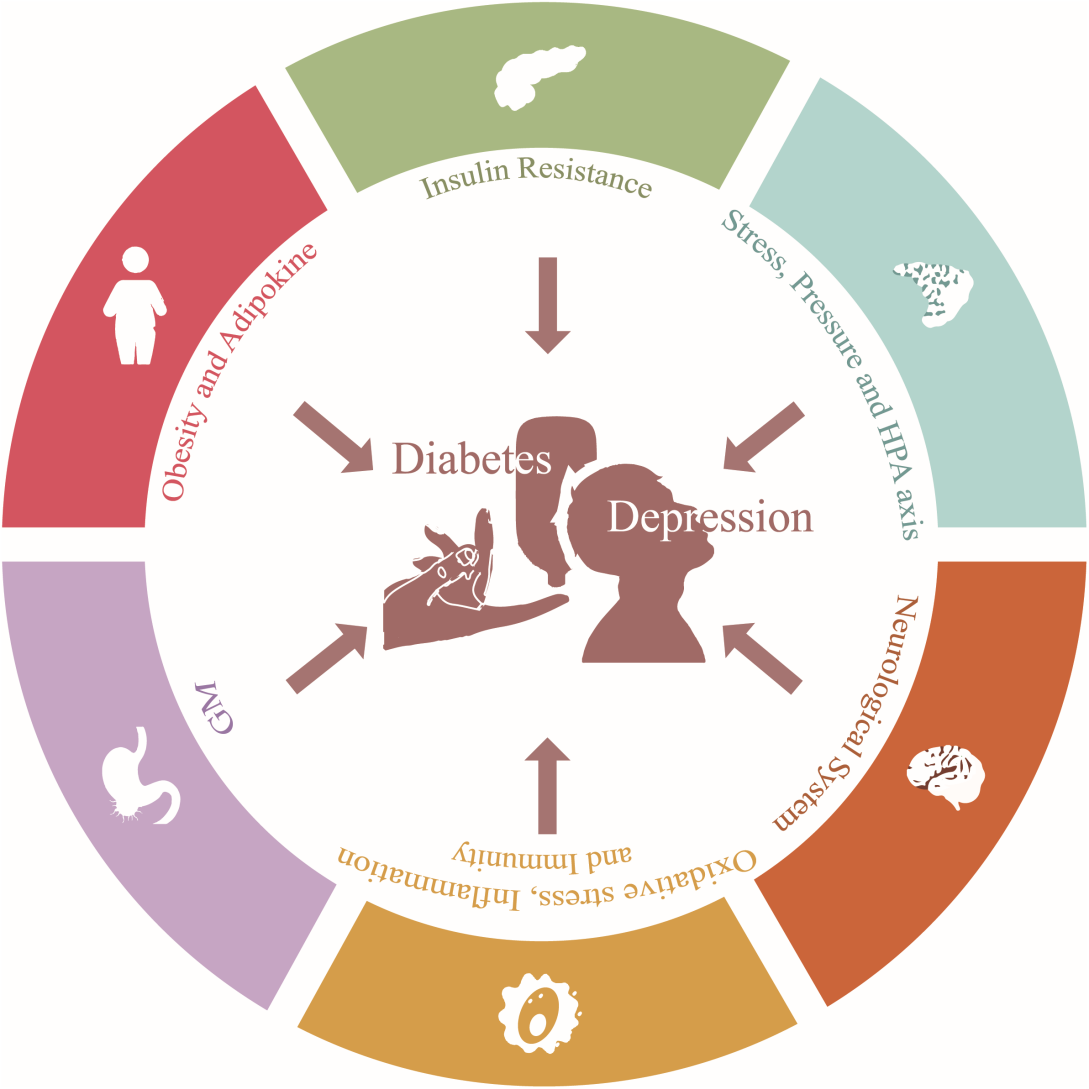
The pathogenesis underlying the comorbidity between diabetes mellitus and depression. HPA, Hypothalamic–Pituitary–Adrenal; GM, Gastrointestinal Microbiome. By Figdraw.

Therefore, it is urgent to discover the exact pathogenesis underlying the comorbidity between depressive disorder and diabetes mellitus and explore alternative drug therapy that is more effective and has fewer adverse effects. Given the significant research investigating the mechanisms and potential drugs for treatment, this article reviews the pathophysiology (Figure 1) and drug alternatives of depression with comorbid diabetes mellitus.

## 2 METHODS

The current review refers to the available literature in PubMed and Web of Science. The critical terms related to diabetes mellitus, depression and drug therapy. Full‐length articles and abstracts were screened for the collection of information included in the paper. We included clinical studies discovering the mechanisms of DM with depression in human subjects and experimental studies exploring the pathology of the comorbidity and drug therapy in animal models. Articles informed by observational studies and clinical trials, review or meta‐analysis articles relevant to the topic, and other publications cross‐referenced for additional published articles were included. Studies were excluded if they failed to discuss the mechanisms and therapy in this field; unpublished data and conference abstracts were also excluded. The main concern in the treatment in this review is natural products for the following two reasons. On the one hand, compared to the traditional therapy against depression and diabetes, such SSRIs, SNRIs (Serotonin and norepinephrine reuptake inhibitors), insulin, metformin and pioglitazone, many natural products were found to have anti‐diabetic and anti‐depressant effects and have an ameliorative effect on complications in DM. SSRIs are clinically used in treating major depressive disorder^28^ and depression in DM but are a risk factor for some complications in DM. On the other hand, insulin resistance, HPA axis, neurological system, GM oxidative stress, inflammation and obesity contributed to the pathology of the comorbidity of DM and depression. Much research has discovered the performance of natural products on these mechanisms. Therefore, we summarize SSRIs, SNRIs, insulin, metformin, pioglitazone and natural products to find potential drugs for comorbidity.

## 3 MECHANISMS

### 3.1 Insulin resistance

Individuals with higher insulin resistance in T2DM display more depressive symptoms, such as irritability, anhedonia, fatigue, and hypersomnia.^19^ Additionally, rats exposed to HDF followed by STZ injection showed induced insulin resistance.^29^ The association between markers of hyperglycemia and insulin resistance and prevalent depressive symptoms was common.^10^ The interaction between insulin and its receptor leads to the stimulation of insulin receptor substrate (IRS) proteins, which then activate two principal insulin signaling pathways: the phosphatidylinositol 3‐kinase (PI3K)‐AKT/protein kinase B (PKB) pathway and the ras‐mitogen‐activated protein kinase (MAPK) pathway. Research findings have indicated that insulin resistance is linked to the IRS and PI3K‐AKT/PKB pathways. The correlation between IRS1 and Langerhans' islets was weakened due to a number of indirect intermediate steps involved in the connection between reduced phosphorylated IRS1 level and destruction of β‐cells.^30^ In diabetic mice and renal cells exposed to high glucose, reduced IRS‐1 levels caused significant up‐regulation of tumor protein 63 (TP63), which demonstrated the potential of TP63 in regulating IRS‐1 and insulin resistance.^31^ Furthermore, PI3K‐generated phosphatidylinositol‐(3,4,5)‐triphosphate facilitated the activation of various serine/threonine kinases dependent on phosphatidylinositols‐(3,4,5)‐triphosphate, particularly AKT/PKB.^32^ Both angptl7 and secreted frizzled‐related protein (sFRP) 4 promoted insulin resistance by inhibiting the activation of Akt.^33^, ^34^ Exercising increased glucose uptake rate in T2DM by PI3K and AS160 activity.^35^

The impairment of Insulin receptors also leads to DM and depression. There are two primary insulin receptor (IR) isoforms, A and B. Insulin‐like growth factor‐2 (IGF‐2) activated IR‐A, which was exclusively expressed by neurons and displayed high binding affinity.^36^ In mice lacking astrocyte IR, brain slices showed impaired dopamine release, and the latter increased depression symptoms. The situation was mainly because astrocytic insulin signaling regulated the phosphorylation of Munc18c and the exocytosis of ATP via syntaxin‐4, which modulated the activity of presynaptic dopaminergic neuronal and the release of dopamine.^37^ Research showed that the minor alleles of both rs2245649 and rs2229429 in the insulin receptor gene (INSR) were risk factors for poor glycaemic control in type 1 diabetes mellitus (T1DM) and were associated strongly with the absence of anti‐insulin antibodies (IAs) in T1DM.^38^ Experimental research revealed that reducing the amount of 5′‐nucleotidase, cytosolic II (NT5C2), and CD36 inhibited insulin signaling by suppressing insulin receptors.^39^ Insulin and insulin‐like growth factor‐1 (IGF‐1) may contribute to antidepressive‐like behavior, yet the antidepressive‐like effect was hindered by JB‐1, an IGF receptor antagonist.^40^ A study demonstrated that DM mice could be induced anxiety‐ and depressive‐like behaviors by knock‐out IR on astrocytes.^37^

### 3.2 Stress and hypothalamic–pituitary–adrenal (HPA) axis

It has been noted that the messed up HPA axis plays a part in the emergence of depression. The boost in serum corticosterone among diabetic patients and rats suggests that the HPA axis is too active.^41^, ^42^, ^43^ Among patients with T2DM, there was an association between low fasting levels of 2‐h C‐peptide and higher levels of midnight cortisol and higher severity of depression.^21^ Research revealed that stressor demands and stress perceptions were associated with depressive symptoms. According to the longitudinal pattern, hair cortisol responded to contextual features related to anticipation, novelty/familiarity, and social evaluative threat.^44^ Another study found that morning cortisol levels were inversely correlated with the frequency of using effective prosocial coping strategies.^45^ Both suggest that although stress increased cortisol levels and depressive symptoms, they followed distinct trajectories.^44^ Depressive symptoms were associated with blunted and exaggerated cortisol responses to and recovery from stress, indicating the high risk of HPA axis dysregulation.^46^ It was found that hsa_circ_0111707, mediated a negative correlation between scores of “demands at work” and “insecurity at work,” was associated with the risk of type 2 diabetes mellitus via sponging miR‐144‐3p.^47^ According to the neuroplasticity hypothesis, glucocorticoids (GC) negatively affected neural development. According to the neurogenesis hypothesis, GC negatively affected neural precursor cell proliferation. This phenomenon was proven by the fact that indirectly, anti‐depressants reversed GC's adverse effects on neural precursor cells via the neuron–noradrenaline–cAMP response element‐binding protein (CREB) pathway and/or the astrocytes–fibroblast growth factor 2 (FGF2) pathway.^48^

The hyperactivity of the HPA axis has an association with DM. In patients with T2DM, GC resistance resulted in the hyperactivity of the HPA axis and hypercortisolism. High GCs led to hyperglycemia. In GC‐sensitive peripheral tissues, the glucocorticoid receptor (GR) regulated glucose production, uptake, and insulin signaling.^49^ Rat hepatocytes lacking GR inhibited hyperglycemia development, indicating that liver‐specific GC antagonists may help control hyperglycemia.^50^ GLP‐1 receptor (GLP‐1R) agonists activated the acute neuroendocrine responses to stress. Diabetes mellitus, as a stressful metabolic situation for cells, induced chronic activation of the HPA axis in the long term, perhaps contributing to insulin resistance.^51^ After inhibiting FoxO1, insulin regulates adrenal steroidogenesis by increasing the expression and activity of steroidogenic factor 1 (SF‐1).^52^

### 3.3 Neurological system

#### 3.3.1 Brain structure and function

**FIGURE 2:**
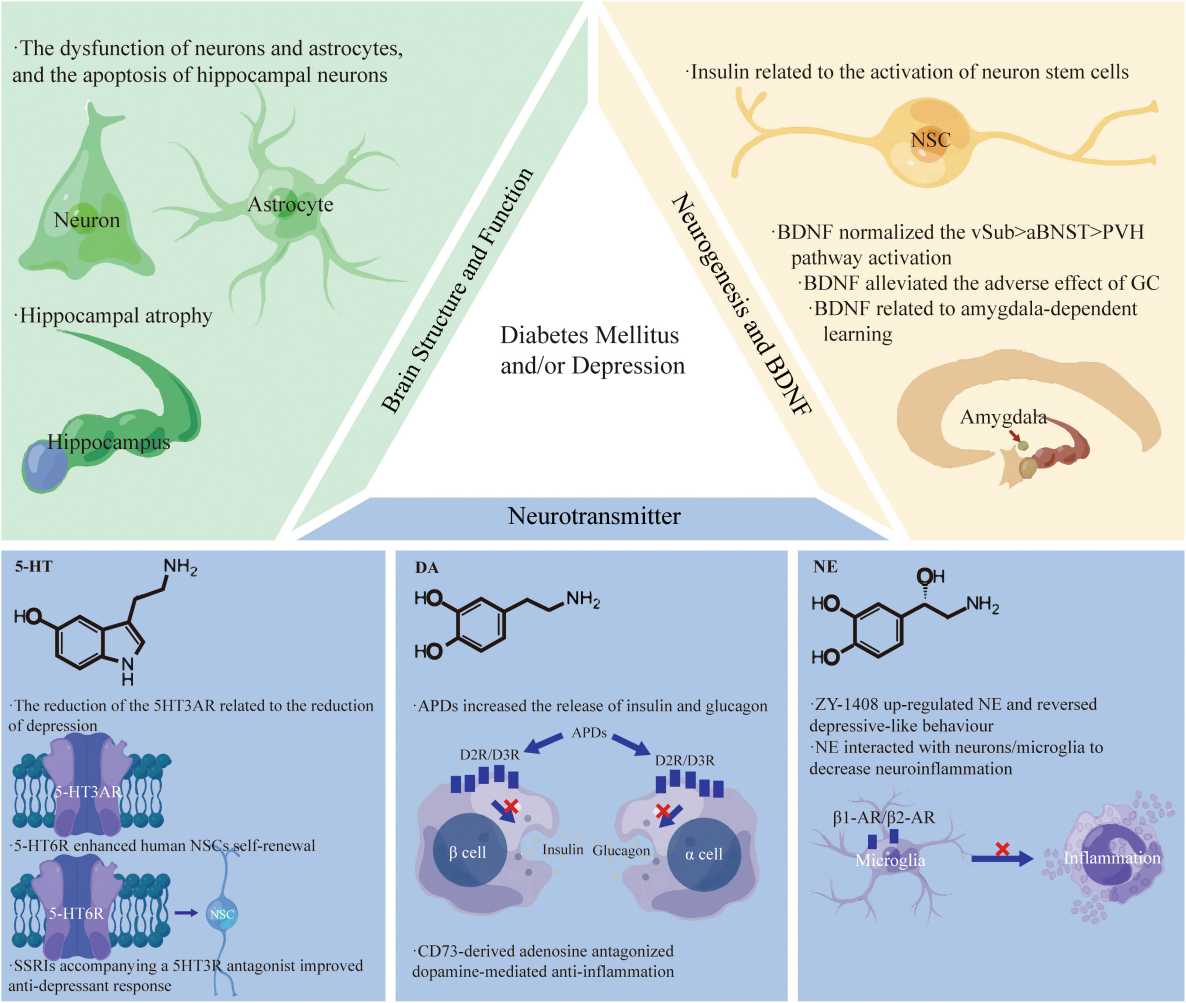
The neurological system, including brain structure and function, neurogenesis, BDNF, and neurotransmitter, are involved in the pathophysiology of diabetes mellitus and depression. APDs, Antipsychotic drugs; DA, Dopamine; NE, Norepinephrine. NSC, Neuron stem cell; SSRIs, Selective serotonin reuptake inhibitors; By Figdraw.

It was proven that there was a close association between abnormality of the fornix, hippocampus and prefrontal cortex and depression with comorbid DM. In T2DM, hippocampal atrophy occurred asymmetrically, with more significant atrophy occurring on the right than the left, probably leading to cognitive impairment.^53^ In the hippocampal CA1 region, diabetes increased the number of apoptotic cells, astrocytes, and mitotic activity and stimulated apoptosis and cell proliferation^54^ (Figure 2). The research found that in mice with T2DM, fewer new neurons were formed in the hippocampus. There was a deficit in the clone formation capacity of neural progenitor cells. In hippocampal neurospheres, insulin and epidermal growth factor receptors decreased.^55^ Similarly, many neuropsychiatric diseases had abnormalities in tissues and organs^56^ like the brain.^57^ For example, MDD also had hippocampal volume reductions, especially for those with more severe depression.^58^ Furthermore, in the depression subject, neurons' and glial cells' volume and number were decreased by 20% to 35% across all hippocampal regions.^59^ The reduction of systemic vascular health, considering diabetes mellitus, had a close relationship with loss of microstructural integrity in the fronix and decline of FA in the frontix and the hippocampal cingulum.^60^, ^61^ The myelin integrity disruption in the fornix was also found in patients with rMDD.^62^ It was revealed that in individuals with T2DM and MDD, cognition and brain structural connectivity were involved in a polygenic risk of T2DM.^63^ It was discovered that the prefrontal–hippocampal circuits originated from the fornix, output fibers to the hippocampus and went back to the prefrontal cortex.^64^

Much research had explored the molecular regulation of the neurological system in neuropsychiatric diseases, such as mitochondrial impairment.^65^ Although the molecular regulation of prefrontal‐hippocampal circuits remains unclear, many studies focused on the relationship between the Glu‐Gln cycle and these brain regions. Glu‐Gln cycle inhibition, anaerobic glycolysis increase, and defects in the lactate‐alanine shuttle may be associated with diabetic depression (DD) development in rats. The latter was also linked to the dysfunction of neurons and astrocytes.^66^ DD rats demonstrated depressive‐like behavior and monoamine neurotransmitter deficiency due to mitophagy‐induced apoptosis of hippocampal neurons through aberrant Glu‐GluR2‐Parkin pathways.^67^ The deep brain stimulation in the frontix could up‐regulate the hippocampus's glucose metabolism by down‐regulating the old mice's extracellular glutamate levels.^68^ In addition, the changes in Glu metabolism in the hippocampus and prefrontal cortex were associated with cognitive impairment and depressive symptoms linked to early life stress.^69^

#### 3.3.2 Neurogenesis and BDNF

An experimental study showed that hyperglycemia in diabetes led to apoptosis and pyroptosis of hippocampal neuron cells, thus resulting in depressive phenotypes.^70^ Islet Antigen‐2 (Ia‐2) loss and over‐expression, which was required for insulin secretion of neurons, induced the proliferation of glia and production of neural stem cells (NSC) from glia by regulating Drosophila insulin‐like peptide 6 (Dilp‐6).^71^ The evolutionarily conserved pseudokinase Tribbles (Trbl) promoted the degradation of Cdc25String to induce the quiescence of G2 NSCs. Trbl inhibited the activation of Akt subsequently to maintain quiescence. But insulin signaling allowed neural stem cells (NSCs) to exit quiescence by overriding repression of Akt and silencing trbl transcription.^72^ The phosphorylation of cAMP response element‐binding protein (CREB) and the CREB target genes expression in the hippocampus was inhibited by a high‐fat diet (HFD). Still, NSC‐derived exosomes (exo‐NSC) induced the CREB recruitment to restore Sirt1, nNOS, BDNF, RelA genes, and Egr3 transcription.^73^ Mob4, Cka, and Hsp83 reactivated NSCs by activating insulin receptor(InR)/PI3K/Akt pathways^74^, ^75^ (Figure 2).

It has been shown that there is increased serum cortisol and miR‐128 and decreased levels of shortened telomeres and BDNF in patients with T2DM and depression.^29^, ^76^ The over‐expression of BDNF promoted the polarization of M2 macrophage, thus repressing diabetes mellitus‐accelerated atherosclerosis (DMAS) development by the inactivation of the STAT3 pathway.^77^ In the intact and diabetic brain, BDNF‐driven hippocampal activity was significant in maintaining the vulnerability towards stress vulnerability, because BDNF over‐expression (BDNFOE) could normalize the ventral subiculum (vSub) > anterior bed nucleus of stria terminalis (aBNST) > paraventricular hypothalamus (PVH) pathway activation.^78^ In addition, the research found that the increase in platelet BDNF levels had significant correction with left amygdala responses, suggesting the importance of BDNF in amygdala‐dependent learning in MDD.^79^ According to the neuroplasticity hypothesis, anti‐depressants and BDNF could alleviate the adverse effects of GC on neural morphology. The phenomenon was because ketamine improved neural morphology through the BDNF–mammalian target of rapamycin (mTOR) pathway^48^ (Figure 2).

#### 3.3.3 Neurotransmitter

Serum serotonin (5‐HT) was found to have a robust correlation with blood glucose and depression.^80^ Depression individuals had higher platelet 5‐HT than the healthy group, whose Hamilton Depression (HAMD) scores were significantly decreased by the administration of SSRIs for 4 weeks.^81^ However, patients with recurrent depressive disorder (RDD) showed higher plasma serotonin concentration and lower platelet serotonin content, which was associated with psychotic symptoms.^82^ The level of 5‐HT in the hippocampus, the expression of the brainstem's 5HT3A receptor, and tryptophan hydroxylase two were found to be reduced in Neuroplastin 65 knock‐out (Np65 KO) mice, displaying reduced depressive‐like behaviors partially.^83^ The administration of SSRIs accompanying a 5‐HT3 receptor (5HT3R) antagonist improved anti‐depressant response.^84^ However, the activation of the 5‐HT6 receptor (5‐HT6R) enhanced human NSC's self‐renewal to induce human cerebral organoids' expansion and folding, and the mice without the 5‐HT6R gene showed depression‐like behaviors.^85^ In addition, the hippocampus in SDT fatty had a significant decrease in 5‐HT concentrations^86^ (Figure 2).

Problems with the brain's dopamine system correlate with DM and depression. In STZ mice, dopaminergic neurotransmission was downregulated in the amygdala.^87^ Dopamine (DA) was α2A‐adrenergic receptors' biased agonist, so antipsychotic drugs (APDs), which blocked DA D2‐like receptors in islets, remarkably increased the release of insulin and glucagon.^88^ The reduction of β‐cell functional insulin secretion was accompanied by the decrease in endocrine dopamine D2 receptor and dopamine D3 receptor.^89^ Furthermore, because inflammation plays a vital role in the pathogenesis underlying diabetes and comorbid depression, which will be mentioned in the following article, CD73‐derived adenosine activated A2A to antagonize dopamine‐mediated anti‐inflammation, thus enhancing inflammation.^90^ Exercise increased DA serum levels, which enhanced the anti‐inflammation of activity in return by inhibiting tumor necrosis factor (TNF) production of splenocytes.^91^ In diabetic septic mice, dopaminergic agonist type‐1 inhibited splenic p65NF‐kB phosphorylation to attenuate systemic inflammation and hyperglycemia.^92^ In addition, HFD exposure reduced TH levels and increased TH phosphorylation at serine 40 in the ventral tegmental area. These effects were associated with insulin resistance, increased tumor necrosis factor‐α levels, oxidative stress, astrogliosis and microglia activation.^93^ Blunted central nervous system insulin receptor signaling through a HF diet could impair DA homeostasis, disrupting cognitive and reward circuitry in regulating hedonic feeding^94^ (Figure 2).

ZY ‐1408 remarkably up‐regulated the hippocampus's norepinephrine (NE) and 5‐HT extracellular concentrations and reversed depressive‐like behavior.^95^ In the spinal cord and brain, remarkable regression of the locus coeruleus (LC)‐NE system was associated with an increase in neuroinflammation and the activation of microglia.^96^ Neurons/microglia may interact with NE via β1‐AR and β2‐AR.^97^ CUMS caused a significant depletion of the norepinephrine transporter (NET).^98^ The interaction between depression and hypertension might be affected by the NET gene differential expression with the target towards TNF‐α and IL‐6.^99^ Chronically elevated NE decreased rates of net glucose uptake in the fetus through insulin resistance^100^ (Figure 2).

### 3.4 Oxidative stress, inflammation and immunity

A wide variety of dysfunctions in oxidative stress, inflammation, and immunity affect the comorbidity between depression and DM. A high level of oxidative stress, expressed by more elevated serum PAB (prooxidant‐antioxidant balance) values and high serum C‐reactive protein (CRP) levels, was found to be associated with depressive symptoms.^101^, ^102^, ^103^ MDD was associated with lower non‐enzymatic antioxidants, rising pro‐inflammatory pathways' activity and other apoptotic mediators' activity, like Caspase‐3, resulting in neuronal death.^104^, ^105^ Because the brain increased oxygen consumption and lipid content and decreased anti‐oxidative defense, it was more vulnerable to oxidative stress (OS), contributing to neurodegeneration.^104^ In addition, the higher plasma NO levels correlated with MDD^106^, ^107^, ^108^ and depressive symptoms, such as fatigue, weight loss, psychomotor retardation, sexual dysfunction, irritability, and indecisiveness.^107^ Consistently, NO blockers were proven to have anti‐depressant effects in MDD patients.^107^ Furthermore, the nitric oxide synthase (NOS) genotype may be necessary in mediating peripheral NOx‐ concentration.^108^ NOS, especially nNOS and eNOS, contributed to the damage of uterine tissue in DM patients.^109^

Consistent with that the expression of pro‐inflammatory cytokines like interleukin‐6 (IL‐6) and tumor necrosis factor–α (TNF‐α) in T2DM rats increased.^29^ Patients with DM and depressive disorder also suffered from a higher level of the systemic immune‐inflammation (SII) index and a lower level of soluble tumor necrosis factor‐like weak inducer of apoptosis (sTWEAK).^20^, ^110^ In diabetes‐depressed conditions, hippocampus microglia chemokine I receptor (CX3CR1) expression and several pro‐inflammatory factors secretion, especially TNF‐α, IL‐6, IL‐8, and IL‐1β were up‐regulated.^111^ Consistent with these researches, the TNFα inhibitor etanercept alleviated impaired recovery induced by prolonged learned helplessness and improved the blood–brain barrier (BBB).^112^ In addition, the evidence that the visual cortex anatomy in depressed patients was affected by a genetic variation, which selectively raised TNF‐α expression, supported the notion that the anatomical changes were partially regulated by the genetic determinants within the activity of inflammation.^113^ Similarly, T2DM has also been found to have a high frequency of the G allele in the TNF‐α ‐308G/A polymorphisms.^114^ Research also found that DN development in T2DM possibly resulted from the synergies of T‐lymphocytes and the TNF‐α signaling pathway.^115^

In MDD, there was an increase in the Th17:Treg ratio and elevated circulating T helper 17 (Th17) cell percentage.^116^ Moreover, patients with a higher risk of suicide showed a robust increase in Th17 cells.^117^ The microbiota was shown to increase Th17 cell production to promote depressive‐like behaviors by producing the quorum sensing molecule autoinducer‐2 (AI‐2) and promoting the production of serum amyloid protein‐1 (SAA1) and SAA2 by the host.^118^ In T2DM, disease‐predictive inflammation was promoted by long‐chain acylcarnitine combined with compromised β oxidation through activating Th17 inflammation.^119^ In T1DM with an SNP (rs12150220) in NLRP1, the levels of IL‐17 and memory Th17 cells were decreased in peripheral blood mononuclear cells, proving that NLRP1 is a potential therapy target.^120^ In lean T2D patients, Th17‐like CD4 + CXCR5+ T cells increased, which was associated with the positivity of autoantibody.^121^ The memory T and B cells induced an up‐regulation of antibodies towards glutamic acid decarboxylase 65 (GAD65) of peripheral blood mononuclear cells (PBMCs) in T1DM. The memory T and B cells were selectively activated by EVs in PBMCs and human islet EVs.^122^

### 3.5 Gastrointestinal microbiome (GM)

Mechanism studies verified that the gastrointestinal microbiome is linked to the development of DM and depression. There was an association between a smaller decrease in *Bacteroides fragilis* and a higher increase in insulin sensitivity.^123^ Intermittent hypoxia (IH) exposures induced changes in GM, increased gut permeability, and altered plasma exosome cargo, the latter causing adipocyte dysfunction (increased IR).^124^ In overweight and obese participants, the associations between trimethylamine N‐oxide (TMAO) changes and increased insulin sensitivity and glycemia were regulated by dietary fat intake.^125^ Dorea, Oscillospira, and Ruminococcus, which fermented polysaccharide into short‐chain fatty acids (SCFAs), was abundant. Increased inflammation exhibited a low level of Turicibacter and a high level of Lactococcus, all of which suggest that the development of obesity may result from the alteration of gut microbiome via increasing systemic inflammation and insulin resistance.^126^ PHZ supplementation alleviates insulin resistance and attenuates gut microbiota alterations induced by HFD.^124^ The administration of fecal microbiota transplantation (FMT) improved insulin resistance and repaired impaired islets by inhibiting β‐cell apoptosis.^127^

In the hippocampus, pro‐inflammatory cytokines were regulated by GM through the dysfunction of the microbiota–gut–brain axis, aggravating the phenotypes of anxiety and depression.^128^ It was generally recognized that probiotics exerted anti‐depressant and anxiolytic effects, but these effects were minor and concentrated on microbial diversity profile.^129^, ^130^, ^131^ Therefore, the pooled results of probiotics should be confirmed by trials with clinical samples.^130^

### 3.6 Obesity and adipokine

**FIGURE 3:**
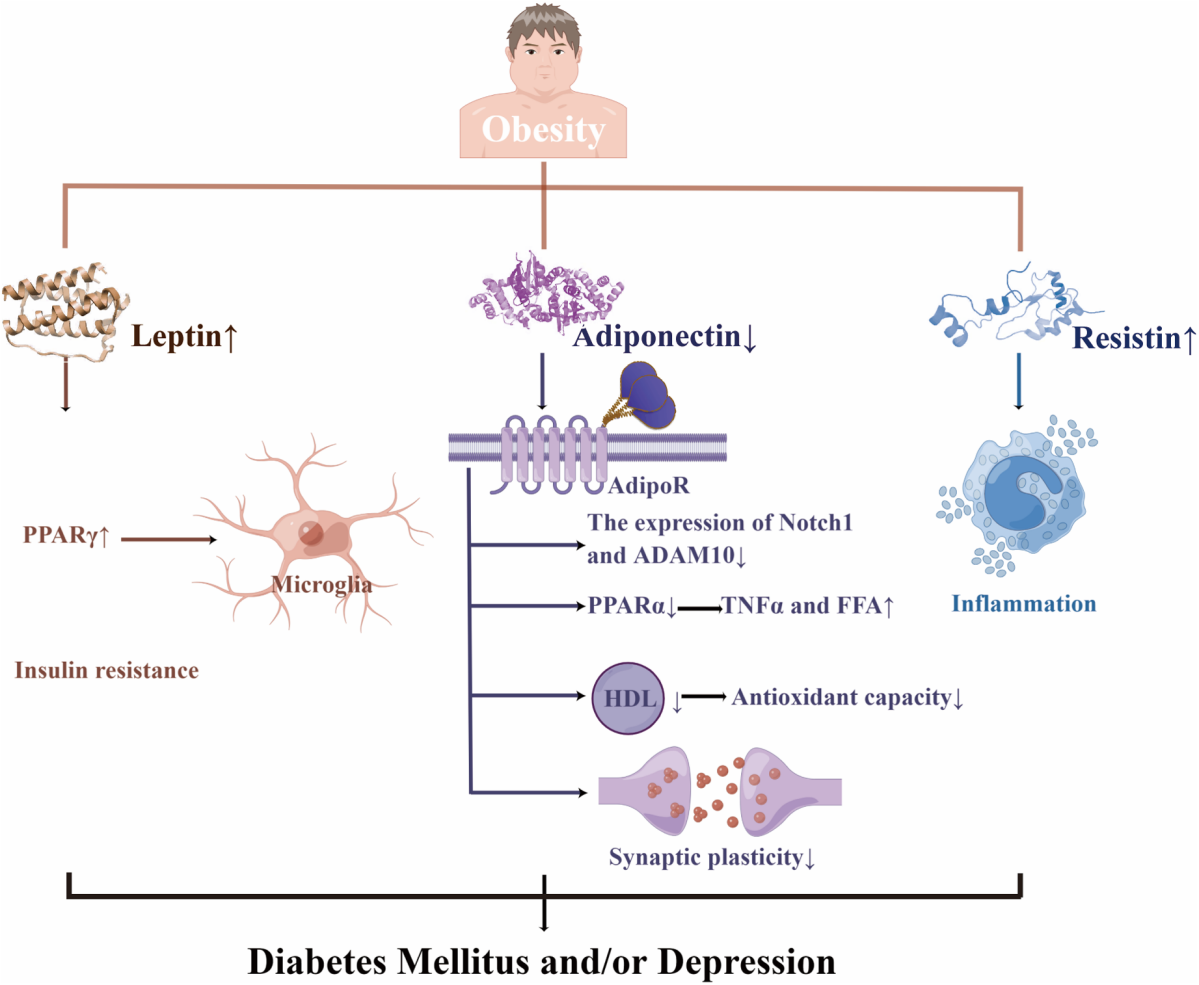
Obesity may lead to diabetes mellitus and depression through reduced adiponectin and increased leptin and resistin. Leptin increased the expression of PPARγ to up‐regulate the activation phenotype of microglia, and related to insulin resistance. The reduction of adiponectin contributed to down‐regulated Notch 1 and ADAM10, increased PPARα, declined HDL and impaired synaptic plasticity. Resistin promoted inflammation. AdipoR, Adiponectin receptor; FFA, Free fatty acid; HDL, High‐density lipoprotein; TNF‐α, Tumor necrosis factor‐α. By figdraw.

It was pointed out that central adiposity may mediate the relationship between uncontrolled diabetes and depression.^11^ Depression had synergistic effects with obesity on diabetes incidence in Chinese adults.^132^ Likewise, patients with diabetes and obesity had an increased risk of depression.^133^ Moreover, another research found that the link between insulin resistance and depression possibly resulted from the over‐adjustment of central obesity.^10^ Furthermore, it was demonstrated that obesity, depression, and DM were associated with low adiponectin levels, high leptin levels, leptin resistance, and high resistin levels^134^, ^135^, ^136^, ^137^, ^138^, ^139^, ^140^ (Figure 3).

Adiponectin could increase fat metabolism, control insulin sensitivity, modify homeostasis, and regulate glucose tolerance to protect individuals from diabetes.^135^ Not only lower antioxidant capacity but also a worse effect of type 2 diabetes mellitus on high‐density lipoprotein (HDL) functionality by rising APO AI, particle size, and cholesterol content, correlated with higher adiponectin.^141^ Adiponectin knock‐out (Adp−/−) mice increased concentrations of TNFα and free fatty acid (FFA) by down‐regulating peroxisome proliferator‐activated receptor (PPAR)α levels, thus leading to insulin resistance.^142^ Adiponectin receptors (AdipoR) 1 and AdipoR2, respectively had a region‐specific effect on anxiety‐like behavior and fear memory extinction. This situation was proven by AdipoR1/2‐dependent synaptic plasticity modulation as well as neuronal excitability.^143^ Chronic stress‐induced hippocampal neurogenesis impairment and cognitive dysfunction may partially result from the inhibition of the Adiponectin‐Notch pathway, which can be reversed by physical exercise. Adiponectin increased Notch1 and ADAM10^144^ (Figure 3).

Ob/ob mice, which are Leptin‐related gene‐deficient mice, showed depressive symptoms after CUMS, possibly by reducing the expression of PPARγ to down‐regulate the activation phenotype of microglia.^145^ The reduction of leptin was associated with alleviating depressive symptoms in obese teenagers after weight loss therapy.^146^ Moreover, the Jackson heart study revealed that insulin resistance was crucial in mediating the correlation between leptin and T2DM.^147^ Endoplasmic reticulum (ER) stress, which was observed to inhibit leptin signaling, and the saturable leptin signaling pathways were involved in developing leptin resistance^148^ (Figure 3).

In the high‐sucrose diet group, anxiety symptoms correlated with metabolic syndrome, which showed hyperglycemia and the reduction of leptin and resistin.^149^ The association between high resistin and reduced T2DM survival may partially result from the pro‐inflammatory nature of resistin.^150^ Hyperglycemia in obesity and DM might enhance the expression of resistin from human mononuclear cells, and resistin might impair insulin sensitivity and promote systemic inflammation in return^151^ (Figure 3).

### 3.7 Metabolic problems due to atypical antipsychotics

In atypical antipsychotic‐naive patients, the T2DM prevalence was 2.9%.^152^ According to a systematic review and meta‐analysis, all antipsychotics were significantly associated with more weight‐gain and a higher risk for a ≥ 7% clinically relevant weight‐gain.^153^ Olanzapine lowered high‐density lipoprotein cholesterol levels. It increased blood prolactin levels and body weight.^154^ Also, in bipolar disorder, olanzapine worsened HbA1c, weight gain and total cholesterol. In the bipolar depression group, olanzapine+fluoxetine worsened total cholesterol and weight gain.^155^ 58.3% of registered clozapine clinic attenders were metabolic syndrome. 79.6% were overweight or obese. 46.6% had elevated fasting blood glucose and 55.2% had elevated blood triglycerides.^156^ The clozapine‐treated group had higher level of diabetes mellitus, diagnosed hypertension and dyslipidemia.^157^ Risperidone‐treated obese mice exhibited increased insulin resistance and glucose intolerance but decreased serum insulin levels. Risperidone could increase serum inflammatory cytokines, reduce pancreatic antioxidant enzyme activity and causes β‐cell damage. Moreover, In the risperidone group, impaired glucose tolerance, increased IR and decreased IS were associated with reduced GLUT4 expression and Akt phosphorylation in insulin signaling.^158^ Potentially clinically significant elevations in clinical chemistry values included triglycerides.^159^ Quetiapine proved to be a relatively safe drug with the most common side effects: headache, somnolence, gastric upset, weight gain, and increased triglyceride levels.^160^

## 4 TREATMENT

### 4.1 SSRIs

According to an observational study, sertraline was conducive to ameliorating depression, and had no aggravating effect on HbA 1c in patients with T2DM.^28^ Likewise, FLX exerted an ameliorative impact on depressive phenotypes and systemic glucose dyshomeostasis, mainly because it down‐regulated PPARγ and adipose triglyceride lipase (ATGL) in visceral white adipose tissue (vWAT), decreased leptin and up‐regulated insulin signaling in the same tissue.^161^ Moreover, SSRIs had been found to reduce the risk of mortality among metabolic syndrome, including diabetes mellitus, complicating depression.^162^, ^163^ Polypharmacy with antipsychotics and psychotropics in bipolar disorder is associated with an increased risk of diabetes mellitus. Although SSRIs mono‐class therapy had an association with a lower risk of DM, the risk of multi‐drug combinations containing SSRIs was relatively high and largely depended on what the other drugs in that combination were.^164^ In skeletal muscle tissue, SSRIs could exert influence on the metabolism of energy, structure properties and electrical muscle activity.^165^ Therefore, reducing the administration of SSRIs could moderate the risk of first falls and fractures among individuals who have diabetes.^166^ Multivariate analysis suggested that SSRIs were the risk factor for CAN (cardiovascular autonomic neuropathy) in T1DM.^167^ In summary, SSRIs have the ability to ameliorate depressive‐like behaviors in DM without exacerbating glucose dyshomeostasis, but are a risk factor for some complications in DM (Table 1).

**TABLE 1:** The effects of drugs on diabetes mellitus and depression.

Natural compounds	Mechanisms and targets	Experiment type	Experiment subject	Insulin resistance	Neurological system	Oxidative stress, inflammation and immunity	Obesity and adipokine	Reference
Selective serotonin reuptake inhibitors	Down‐regulated PPARγ and ATGL in vWAT	In vivo	C57BL/6N mice				√	34198047
	Decreased leptin	In vivo	C57BL/6N mice				√	34198047
	Up‐regulated insulin signaling	In vivo	C57BL/6N mice	√				34198047
	Alter energy metabolism, structure properties and electrical muscle activity	In vivo	Patients and rodents					30196103
Insulin	Modulate the dynamic synaptic transmission and plasticity in the hippocampus of the mouse	In vivo	C57BL/6 mice		√			31146008
Metformin	Corrected abnormal glutamatergic transmission	In vivo	C57BL/6 mice		√			33114529
	Decreased circulating branched‐chain amino acids levels to favor serotonergic neurotransmission in the hippocampus	In vivo	C57Bl6/j mice		√			31160539
	Regulated Lcn‐2 and inflammation‐related molecules expression level	In vivo	C57BL/6 mice			√		31977586
	Enhanced the expression of BDNF by activating AMPK/CREB‐mediated histone acetylation	In vivo	C57BL/6J mice		√			31521867
Pioglitazone	Ameliorated upregulation of ventral hippocampal GFAP, the HFD‐induced glucose‐metabolic dysfunctions	In vivo	C57BL/6N	√				34022606
	Reversed the abnormality of the ventral hippocampal CA1 GFAP‐immunoreactive astrocytes				√			
	Activated the microglia to decrease the susceptibility of stressed ob/ob mice to develop depression	In vivo	wt mice or ob/ob mice		√			32422516
	Improved the imbalance of M1 and M2 inflammatory cytokines	in vitro	Murine N9 microglial cells			√		27716270
	Increased neural apoptosis via up‐regulating the PI3K/AKT pathway and down‐regulating the JNK/p38 pathway	In vivo	C57BL/6 mice		√			29359338
	Modulated NF‐κB/IL‐6/STAT3 and CREB/BDNF pathways	In vivo	C57BL/6 mice					28595081
Hydrogen sulfide	Reduced iron deposition and oxidative stress	In vivo	C57BL/6J mice			√		33945828
	Increased the expression of GPX4 and SLC7A11							
	Modulated Sirt6							
	Upregulated the expressions of BDNF and p‐TrkB proteins	In vivo	SD rats		√			31660632
	Activated the PI3K/AKT pathway	In vivo	SD rats	√	√			34087334
	Reversed hippocampal neurogenesis							
	Reduced malondialdehyde and 4‐hydroxynonenal	In vivo	SD rats			√		25932716
	Elevated superoxide dismutase and reduced glutathione							
Cannabidiol	Increased 5‐HT, NA and/or DA	In vivo	Wistar rats	√	√			32360935
	Increased weight gain and the insulin levels							
	Interacted with 5‐HT1A, CB1, or CB2 receptor	In vivo	Wistar rats					33464458
Ascorbic acid	Increased insulin and monoamines	In vivo	Albino rats	√		√		28827076
Hesperidin	Ameliorated hyperglycaemia, oxidative stress and inflammation	In vivo	Albino Wistar rats		√	√		25358020
	Enhanced neurogenesis							
	Increased monoamines							
	Activated the Nrf2/ ARE pathway	In vivo	SD rats		√			32982741

### 4.2 Serotonin and norepinephrine reuptake inhibitors (SNRIs)

According to the glucose consumption assay, venlafaxine caffeic acid salt showed strong hypoglycemic activity in human liver cells of HL‐7702, suggesting its potential therapeutic effect for the comorbidity of T2DM and depression.^168^ A retrospective study showed that the mean ratio of HbA1c for Venlafaxine was numerically lower than Citalopram, however there is no statistically significant difference.^169^ A study indicated that the risk of T2DM increased because of the exposure of antidepressants, and that Duloxetine and Venlafaxine were the antidepressants most associated with T2DM.^170^ Venlafaxine hydrochloride markedly elevated the consumption of glucose in the glucose consumption test in vitro.^171^ Results from a survey aimed at psychiatrists illustrated that duloxetine was widely applied in fibromyalgia and DM due to its alleviation of neuropathic pain.^172^ However, the administration of SNRIs, tricyclic antidepressants, SSRIs, and other antidepressants was associated with increased risk of diabetes.^173^ Furthermore, treatment with SSRIs or SNRIs correlated with a rise in the risk of T2DM, and this association intensified with increasing cumulative dose, duration of use and average daily dose.^174^, ^175^

### 4.3 Insulin

According to an observational study and meta‐analysis, insulinized patients with DM were found to present remarkable impairment of depressive symptoms.^176^, ^177^, ^178^ The high relation between insulin use and depression resulted from two conditions. On the one hand, it was revealed that adolescents with T1DM were much more likely to reject insulin use because of diabetes distress and reduced self‐care.^179^, ^180^ On the other hand, patients with diabetes mellitus and depression suffered from the uneffective utilization of insulin. Insulin resistance as a cross‐disorder mechanism contributed to the higher somatic comorbidity and shortened lifespan in diabetes and depression. Compared to healthy people, elevated insulin was found in patients with MDD.^181^ Dietary insulin index (DII) and Dietary insulin load (DIL) were discovered to have a positive correlation with the incidence of depression in women.^182^ In addition, depressive symptoms have been implicated in the upregulated production of antibodies against estrogenic insulin in patients with T1DM, which may be partly mediated by depression‐induced inflammation.^183^ However, the improvement of insulin sensitivity could alleviate depression. The progress of glycemic control and reduction of depressive symptoms were related to the higher mealtime insulin bolus score behavior.^184^ The antidepressive effect of insulin and IGF‐1 in rats with DM was mediated by IGF‐1 receptor in brain.^40^ An experimental study revealed that insulin could improve learning and memory by modulating the dynamic synaptic transmission and plasticity in the hippocampus of the mouse, thus improving cognitive impairment^185^ (Table 1). Therefore, insulin administration was not recommended in the therapy of the comorbidity of DM and depression, while the therapy of up‐regulating insulin sensitivity should be recommended.

### 4.4 Metformin

A nationwide population‐based study revealed an association between metformin in continued use and combinations of drugs and decreased rates of incident depression, suggesting a positive effect of metformin on depression rates.^186^ Compared to elderly diabetic patients taking no medication, patients taking metformin showed a lower risk of depression, indicating that metformin was a protective factor against depression in those patients.^187^ Metformin users with T2DM in Korea had a significantly lower prevalence of malignancy and depression.^188^ However, a research found an association between low doses of metformin and lower depression risk in diabetes, while high doses of metformin correlated with increased risk of depression.^189^ In terms of the mechanism underlying the antidepressant effect of metformin, a study found that metformin recovered the glutamatergic transmission, which was elevated in depressive mouse. The result suggested that metformin ameliorated depression via modifying abnormal glutamatergic transmission.^190^ By decreasing circulating BCAA levels in the hippocampus, metformin might serve as an antidepressant in diabetic mice to favor serotonergic neurotransmission in the hippocampus.^191^ Metformin regulated Lcn‐2 and inflammation‐related molecules expression level to down‐regulate depression symptom.^192^ Metformin activated the AMPK‐CREB mediated histone acetylation and up‐regulated the level of BDNF promoter to increase the expression of BDNF, thus alleviating the depression in diabetes. The metformin administration could not only reverse depression in mice but also strengthen the antidepressant effect of fluoxetine in combination with fluoxetin^193^ (Table 1).

### 4.5 Pioglitazone

The pioglitazone treatment could ameliorate the upregulation of ventral hippocampal GFAP (glial fibrillary acidic protein), the glucose‐metabolic dysfunctions and reverse the abnormality of the ventral hippocampal CA1 GFAP‐immunoreactive astrocytes, and alleviate the depression phenotypes.^194^ There were severe impairment of spatial memory, behavioral disorders, an increase in pro‐inflammatory cytokines, and a decrease in antiinflammatory cytokines and Peroxisome proliferator‐activated receptor gamma (PPARγ) in the prefrontal cortex (PFC) and hippocampus in diabetic mice with CUMS. It was revealed that PPARγ activated the microglia, so depression was more likely to develop in stressed ob/ob mice when PPARγ was reduced. As a result, the Pioglitazone, a PPARγ 1 activator, alleviated depressive symptom in mice.^145^ In an in vitro experiment, the administration of pioglitazone alleviated depression by rebalance of M1 and M2 inflammatory cytokines. The imbalanced level of M1 and M2 inflammatory cytokines inhibited nuclear factor kB activation and is expressed in lipopolysaccharide (LPS)‐stimulated N9 microglial cells.^195^ Furthermore, pioglitazone's antidepressant effect was due to an increase of neural apoptosis in the PFC, associated with down‐regulation of the Jun N‐terminal kinase (JNK)/p38 pathway and up‐regulation of the PI3K/AKT pathway.^196^ Pioglitazone had anti‐depressant effect, as indicated by the improvement of behaviors in several tests like open field, elevated plus maze and forced swimming tests.^197^ Pioglitazone's antidepressant effect on depression‐like behaviors was dependent on PPAR‐γ, with the modulation of the nuclear factor kappa B/interleukin 6/signal transducer and activator of transcription 3 (NF‐κB/IL‐6/STAT3) and CREB/BDNF pathways^198^ (Table 1). However, according to a double‐blind, placebo‐controlled trial, pioglitazone was not found to be more effective than placebo at treating depression based on inflammatory and metabolic markers.^199^

### 4.6 Hydrogen sulfide (H2S)

Many mechanisms are involved in the effect of hydrogen sulfide, a gaseous mediator, on depression with comorbid DM. For instance, sodium hydrosulfide (NaHS), a donor of H2S, remarkably down‐regulated the PFC ferroptosis via decreasing oxidative stress and iron deposition. NaHS reduced anxiety‐like and depressive‐like behaviors by modulating sirtuin 6 (Sirt6) to inhibit inflammation and reducing the PFC ferroptosis in the BV2 cells and T1DM.^200^ In the hippocampus of STZ‐induced diabetic rats, BDNF and p‐tropomyosin‐related kinase B (TrkB) proteins were up‐regulated by H2S.^201^ In the hippocampus, NaHS triggered PI3K/AKT pathway and promoted neurogenesis induced by STZ, displaying an anti‐depressant effect.^202^ H2S exerted an ameliorative influence against depression and anxiety by inhibiting oxidative stress in the hippocampus of diabetes^203^ (Table 1). Exogenous H2S inhibited autophagy via activating the PI3K/AKT1 signaling pathway to mitigate diabetes‐induced myocardial fibrosis. Exogenous H2S not only promoted autophagy via activating the BDNF/TrkB pathway to improve diabetic depression but also inhibited autophagy via the Nrf2‐ROS‐AMPK signaling pathway to improve endothelial cell density (ECD) in diabetic rats.^204^

### 4.7 Cannabidiol (CBD)

Many experiments support that the administration of cannabidiol helps alleviate depression and diabetes mellitus. CBD treatment (high dose) not only reduced the depressive symptoms in diabetic rats, as evidenced by the altered level of 5‐HT, NA and/or DA, but also caused a remarkable increase in weight gain and the level of insulin, thus reducing glycemia.^26^ Sub‐chronic treatment with cannabidiol (high dose) induced a mild effect against depression.^27^ The impact of CBD against depression was blocked by the antagonists −5‐HT1A, cannabinoids type‐1 (CB1), or CB2. The effect of CBD against glycemia was blocked by the blockade of CB2 in diabetic animals. These phenomenons indicated that cannabidiol induced different effects through multiple sites of action^205^ (Table 1). CBD caused a strain‐dependant antidepressant‐like effect, as evidenced by the improvement of the tail suspension test in Swiss rats exclusively. CBD exerted a sex‐dependant anti‐depressant effect, as proven by the improvement of the tail suspension test in the male Swiss rats exclusively, and had a time‐dependant antidepressant‐like effect, as evidenced by the effect against depression in male Flinders Sensitive Lines (FSL) rats but an effect with bimodality in female FSL.^206^ In MIN6 as well as Beta‐TC‐6, cells abnormal cannabidiol (Abn‐CBD) down‐regulated apoptosis induced by ER stress via the CREB phosphorylation of β‐cells.^207^

### 4.8 Ascorbic acid (AA)

It has been demonstrated that ascorbic acid could have an ameliorative effect on DM and depression. Ascorbic acid exerted a dose‐dependent influence on reducing the immobility period, hyperglycemia, and hypoinsulinemia.^208^ The treatment of metformin combined with AA was shown to down‐regulate glucose, immobility period, and corticosterone levels, up‐regulate the insulin and monoamine levels and decrease pro‐inflammatory cytokines as well as oxidative stress in diabetic rats with comorbid depression^209^ (Table 1). Also, in CUS‐induced rats, AA and ketamine in single sub‐effective doses have alleviated depressive‐like behavior.^210^ Ascorbic acid not only ameliorated 24 h as well as postprandial glycemia but also down‐regulated blood pressure in T2DM.^211^ Pre‐exercise and postexercise blood pressure (BPs) in patients with T2DM decreased after 6 weeks of Vitamin C supplementation, possibly resulting from improved oxidative stress and NO release.^212^ Glucose transporter 10 (GLUT10) regulated adipogenesis, and prevented mice from HFD‐induced metabolic dysregulation by maintaining AA‐dependent DNA demethylation.^213^

### 4.9 Hesperidin

Many studies indicated that hesperidin has the potential to cure depression and DM. Hesperidin had an anti‐depressant effect in diabetic rats, possibly because it alleviated hyperglycemia, oxidative stress, and inflammation, enhanced neurogenesis, and changed the levels of monoamines in the brain.^214^ Hesperidin alleviated the symptoms of depression and anxiety in diabetic animals via activating the nuclear factor erythroid 2‐related factor 2 (Nrf2)/ARE/Glyoxase 1 pathway^215^ (Table 1). Also, in mTBI‐induced mice, hesperidin reduced depression by decreasing TNF‐α, IL‐1β and malondialdehyde (MDA), and increasing BDNF levels in the hippocampus.^216^ In CUMS‐induced diabetes, hesperidin treatment alleviated depression by inhibiting inflammation and microglia, as evidenced by the decreased expression of IL‐6, IL‐1β, NLRP3, TNF‐a, ASC, and caspase‐1 in the microglia and prefrontal cortex.^217^ Hesperidin protected pancreatic β cells and improved their function in diabetic rats, possibly by inhibiting ER stress as well as oxidative stress, along with the effects against oxidation, inflammation, and apoptosis.^218^ Systolic blood pressure (SBP), mean arterial blood pressure, IL‐6 and hs‐CRP were reduced in the hesperidin group, suggesting that hesperidin may have effects against hypertension and inflammation in T2DM.^219^

## 5 CONCLUSION AND LIMITATION

The potential of this review was presented in the following aspects. First, this review demonstrated that stress contributed to the hyperactivity of the HPA axis, and then resulted in depression, indicating that medical personnel should pay attention to diabetes distress to avoid depression. Second, it was revealed that prefrontal‐hippocampal circuits had structural and functional abnormalities in the comorbidity, suggesting the potential to explore these circuits' molecular regulation. Third, obesity might lead to diabetes mellitus and depression through reduced adiponectin and increased leptin and resistin, indicating the importance of weight control.

To reach the ultimate goal of finding anti‐diabetic and anti‐depressant drug therapy, more efforts should be made to solve limitations and conduct more studies in the future.

First, the performance of drug therapy in terms of the HPA axis and adipokine was insufficient. Before exploring drug therapy in clinical trials, there is an urge to fully discover the drug therapy in comprehensive mechanisms related to comorbidity to avoid their side effects.

Second, although this review explored the alternative therapy for DM with depression based on different natural products, it is also inspiring to discover the treatment from the perspective of different pathophysiological hypotheses. For example, an animal experiment suggested that agmatine could alleviate insulin resistance by remarkably inhibiting T2DM‐induced depression, anxiety, and neuroinflammatory markers in rats.^29^ Animal research found that Quercetin and Rutin improve glucose control and alleviate depressive symptoms in rats mainly by inhibiting the HPA axis response.^43^ From the oxidative stress perspective, date seed extract could reduce blood glucose and oxidative stress to prevent the brain regions from lipid peroxidation products, thus exhibiting an anxiolytic effect.^220^ Metformin attenuated depressive symptoms mainly because it increased brain‐derived neurotrophic factor (BDNF) by up‐regulating the histone acetylation and the BDNF promoter.^193^ From the aspect of GM, the banana starch diet ameliorated depression in diabetic rats mainly due to the improvement of the gut‐microbiota‐brain axis.^221^ In adipokines, plant and marine sources of n‐3 PUFAs have been proved to increase adiponectin and decrease leptin levels in patients with type 2 diabetes.^222^

Third, many animal models and cell models were discovering the comorbidity of DM and depression. However, effective models have not been utilized generally. Therefore, it is necessary to establish effective models in vivo and in vitro to represent the uniqueness of the comorbidity.

There are some weaknesses worth noting in this study. First, since this review is not a systematic review, it cannot discover the comprehensive mechanisms of the comorbidity of DM and depression. Second, the main subjects of literature in this review are animals, leading to the decreased exploration of mechanisms and drug therapies in human subjects. Third, evidence on the antidepressant effects of exercise and diet therapy is increasing, which also played an important role in the treatment of comorbidity of depression and metabolic disturbances. However, this review only included the drug therapy.

Experiments in vivo and in vitro had discovered many meaningful findings in this field. Their scientific value gave researchers further insight into the mechanisms and treatment of comorbidity, thus providing an important theoretical basis. However, there are still some challenges faced by this field. The generally recognized standards of models in vivo and in vitro have not been built up. To find out the well‐recognized models, research should be conducted to identify the critical characteristics of the main models regarding anatomy and physiology of the brain, diabetes mellitus modeling, depression modeling and implementation aspects. In addition, It is necessary to carry out more experiments and research on human subjects to confirm the findings in animal and cell models. Due to the anti‐diabetic and anti‐depressant effects of drug therapy, it is worthwhile to find out the translation value of these experiments in bedside research on human subjects. Therefore, it is of great help to establish large‐scale cohorts of people with DM and depression. The longitudinal follow‐up could investigate the history, mechanisms, treatment response, and prognosis. The underlying pathogenesis could be uncovered after the application of the high throughput techniques in omics.

Researchers should pay much attention to the comorbidity of DM and depression regarding its high prevalence and social burden. Considering that most of the reviews did not provide a thought‐provoking and comprehensive insight into the interface of DM and depression, we summarized the mechanisms underlying the comorbidity and drug therapy with anti‐diabetic and anti‐depressant effects. Previous studies have deeply explored and summarized the contribution of insulin resistance, neurotransmitters, oxidative stress and inflammation towards the comorbidity. This review also summarized numerous studies about stress, the HPA axis, prefrontal‐hippocampus circuits and adipokines. It was found that prefrontal‐hippocampal circuits played a critical role in the comorbidity as a result of dysfunction of the Glu‐Gln cycle. Weight control is significant because of the reduced adiponectin and increased leptin and resistin in comorbidity. But what internal and external elements can trigger the molecular pathways and how the bioactive compounds interact remain unclear in comorbidity. The genetic background should be explored to get further insight into the environmental factors, molecular pathways and effective targets. Since most of the evidence came from animal and cell studies, more research such as clinical research in human subjects, meta‐analysis, omics technologies, and network pharmacology should be conducted. In addition, although the medicines displayed in this review could expand the potential therapy region, exploring their mechanisms of action was recently based on animal experiments. Conducting a large‐scale cohort is a good way to investigate the natural history, pathogenesis and treatment response. We strongly advise that experiments on human subjects should be conducted to establish a novel and effective pharmacological therapy.

## AUTHOR CONTRIBUTIONS

Sixin Li, Dong Yang and Xuhui Zhou discussed the subject matter, drafted the manuscript and prepared the figures. Lu Chen, Lini Liu, Ruoheng Lin, Xinyu Li, Ying Liu and Huiwen Qiu collected the related references and participated in the critical revision of the article. Hui Cao, Jian Liu and Quan Cheng designed, revised and funded the review. All authors contributed to this manuscript. All authors read and approved the final manuscript.

## FUNDING INFORMATION

This study was supported by the Hunan Provincial Natural Science Foundation of China (NO.2023JJ40362 and NO.2022JJ30451), Changsha City Natural Science Foundation of China (NO. kq2208103), National Natural Science Foundation of China (NO.82104793) and Hunan Youth Science and Technology Talent Project (NO.2022RC1226).

## CONFLICT OF INTEREST STATEMENT

The authors declare no conflict of interest.

## Data Availability

Data sharing is not applicable to this article as no new data were created or analyzed in this study.

## References

[1] Huang CJ , Hsieh HM , Tu HP , Jiang HJ , Wang PW , Lin CH . Major depressive disorder in patients with type 2 diabetes mellitus: prevalence and clinical characteristics. J Affect Disord. 2018;227:141‐148. doi:10.1016/j.jad.2017.09.044 29073576

[2] Jaworski M , Panczyk M , Sliwczynski A , et al. Severe depressive episode with psychotic symptoms and type 2 diabetes: a 2010‐2017 longitudinal study. Med Sci Monit. 2019;25(1760–1768):1760‐1768. doi:10.12659/MSM.913356 30846676 PMC6419531

[3] Felix HC , Andersen JA , Willis DE , Malhis JR , Selig JP , McElfish PA . Control of type 2 diabetes mellitus during the COVID‐19 pandemic. Prim Care Diabetes. 2021;15:786‐792. doi:10.1016/j.pcd.2021.06.012 34246614 PMC8449252

[4] GBD 2019 Mental Disorders Collaborators . Global, regional, and national burden of 12 mental disorders in 204 countries and territories, 1990‐2019: a systematic analysis for the global burden of disease study 2019. Lancet Psychiatry. 2022;9:137‐150. doi:10.1016/S2215-0366(21)00395-3 35026139 PMC8776563

[5] COVID‐19 Mental Disorders Collaborators . Global prevalence and burden of depressive and anxiety disorders in 204 countries and territories in 2020 due to the COVID‐19 pandemic. Lancet. 2021;398:1700‐1712. doi:10.1016/S0140-6736(21)02143-7 34634250 PMC8500697

[6] Chen C . Recent advances in the study of the comorbidity of depressive and anxiety disorders. Adv Clin Exp Med. 2022;31(355–358):355‐358. doi:10.17219/acem/147441 35394125 PMC13539099

[7] Rajkumar RP . Comorbid depression and anxiety: integration of insights from attachment theory and cognitive neuroscience, and their implications for research and treatment. Front Behav Neurosci. 2022;16:16. doi:10.3389/fnbeh.2022.1104928 PMC9811005 36620859

[8] Tanaka M , Szabo A , Vecsei L . Integrating armchair, bench, and bedside research for behavioral neurology and neuropsychiatry: editorial. Biomedicine. 2022;10:10. doi:10.3390/biomedicines10122999 PMC9775182 36551755

[9] Saiz‐Vazquez O , Gracia‐Garcia P , Ubillos‐Landa S , et al. Depression as a risk factor for Alzheimer's disease: a systematic review of longitudinal meta‐analyses. J Clin Med. 2021;10:10. doi:10.3390/jcm10091809 PMC8122638 33919227

[10] Geraets AFJ , Kohler S , Muzambi R , et al. The association of hyperglycaemia and insulin resistance with incident depressive symptoms over 4 years of follow‐up: the Maastricht study. Diabetologia. 2020;63:2315‐2328. doi:10.1007/s00125-020-05247-9 32757152 PMC7527373

[11] Dona AC , DeLouize AM , Eick G , et al. Inflammation and central adiposity as mediators of depression and uncontrolled diabetes in the study on global AGEing and adult health (SAGE). Am J Hum Biol. 2020;32:e23413. doi:10.1002/ajhb.23413 32222050

[12] Carr AL , Sluiman AJ , Grecian SM , et al. Depression as a risk factor for dementia in older people with type 2 diabetes and the mediating effect of inflammation. Diabetologia. 2021;64:448‐457. doi:10.1007/s00125-020-05301-6 33064180 PMC7801357

[13] Ravona‐Springer R , Heymann A , Lin HM , et al. Increase in number of depression symptoms over time is related to worse cognitive outcomes in older adults with type 2 diabetes. Am J Geriatr Psychiatry. 2021;29:1‐11. doi:10.1016/j.jagp.2020.09.022 33127316 PMC7771631

[14] Lee HM , Yang YC , Chen SF , Hsu CY , Shen YC . Risk of hyperglycemic crisis episode in diabetic patients with depression: a nationwide population‐based cohort study. J Diabetes Complications. 2020;34:107509. doi:10.1016/j.jdiacomp.2019.107509 31864898

[15] Wang HQ , Wang ZZ , Chen NH . The receptor hypothesis and the pathogenesis of depression: genetic bases and biological correlates. Pharmacol Res. 2021;167:105542. doi:10.1016/j.phrs.2021.105542 33711432

[16] Battaglia S , Cardellicchio P , Di Fazio C , Nazzi C , Fracasso A , Borgomaneri S . Stopping in (e)motion: reactive action inhibition when facing valence‐independent emotional stimuli. Front Behav Neurosci. 2022;16:998714. doi:10.3389/fnbeh.2022.998714 36248028 PMC9561776

[17] Santana‐Santana M , Bayascas JR , Gimenez‐Llort L . Sex‐dependent signatures, time frames and longitudinal fine‐tuning of the marble burying test in normal and AD‐pathological aging mice. Biomedicine. 2021;9:9. doi:10.3390/biomedicines9080994 PMC8391620 34440198

[18] Lu Y , An T , Tian H , et al. Depression with comorbid diabetes: what evidence exists for treatments using traditional Chinese medicine and natural products? Front Pharmacol. 2020;11:596362. doi:10.3389/fphar.2020.596362 33568996 PMC7868339

[19] Brouwer A , van Raalte DH , Lamers F , et al. Insulin resistance as a marker for the immune‐metabolic subtype of depression. J Affect Disord. 2021;295:1371‐1376. doi:10.1016/j.jad.2021.08.151 34565592

[20] Wang J , Zhou D , Dai Z , Li X . Association between systemic immune‐inflammation index and diabetic depression. Clin Interv Aging. 2021;16:97‐105. doi:10.2147/CIA.S285000 33469277 PMC7810592

[21] Sang YM , Wang LJ , Mao HX , Lou XY , Zhu YJ , Zhu YH . Correlation of lower 2 h C‐peptide and elevated evening cortisol with high levels of depression in type 2 diabetes mellitus. BMC Psychiatry. 2020;20:490. doi:10.1186/s12888-020-02901-9 33023555 PMC7539383

[22] Lesiewska N , Borkowska A , Junik R , Kaminska A , Jaracz K , Bielinski M . Consequences of diabetes and pre‐diabetes and the role of biochemical parameters of carbohydrate metabolism for the functioning of the prefrontal cortex in obese patients. Front Biosci (Landmark Ed). 2022;27(76):76. doi:10.31083/j.fbl2703076 35345308

[23] Li HQ , Chi S , Dong Q , Yu JT . Pharmacotherapeutic strategies for managing comorbid depression and diabetes. Expert Opin Pharmacother. 2019;20:1589‐1599. doi:10.1080/14656566.2019.1622090 31149850

[24] van der Feltz‐Cornelis C , Allen SF , Holt RIG , Roberts R , Nouwen A , Sartorius N . Treatment for comorbid depressive disorder or subthreshold depression in diabetes mellitus: systematic review and meta‐analysis. Brain Behav. 2021;11:e01981. doi:10.1002/brb3.1981 33274609 PMC7882189

[25] Kumar PR , Chatterjee A;JPB , Patnaik S . Effect of sertraline as an add‐on therapy in T2DM patients with comorbid depression: an open label randomized controlled trial. Indian J Endocrinol Metab. 2019;23:357‐362. doi:10.4103/ijem.IJEM_67_19 31641639 PMC6683678

[26] Chaves YC , Genaro K , Stern CA , et al. Two‐weeks treatment with cannabidiol improves biophysical and behavioral deficits associated with experimental type‐1 diabetes. Neurosci Lett. 2020;729:135020. doi:10.1016/j.neulet.2020.135020 32360935

[27] de Morais H , Chaves YC , Waltrick APF , et al. Sub‐chronic treatment with cannabidiol but not with URB597 induced a mild antidepressant‐like effect in diabetic rats. Neurosci Lett. 2018;682:62‐68. doi:10.1016/j.neulet.2018.06.006 29885450

[28] Padmapriya C , Pushkarapriya S , Shanmugapriya N , Sushmitha KP , Karthik S , Rajanandh MG . Effect of sertraline in patients with newly diagnosed depression and type 2 diabetes mellitus or hypertension: an observational study from South India. Diabetes Metab Syndr. 2020;14:1065‐1068. doi:10.1016/j.dsx.2020.06.059 32645649

[29] Kale M , Nimje N , Aglawe MM , Umekar M , Taksande B , Kotagale N . Agmatine modulates anxiety and depression‐like behaviour in diabetic insulin‐resistant rats. Brain Res. 2020;1747:147045. doi:10.1016/j.brainres.2020.147045 32758481

[30] Atmodjo WL , Larasati YO , Jo J , Nufika R , Naomi S , Winoto I . Relationship between insulin‐receptor substrate 1 and Langerhans' islet in a rat model of type 2 diabetes mellitus. In Vivo. 2021;35:291‐297. doi:10.21873/invivo.12258 33402476 PMC7880789

[31] Liang S , Nayak BK , Vogel KS , Habib SL . TP63 is significantly upregulated in diabetic kidney. Int J Mol Sci. 2021;22:22. doi:10.3390/ijms22084070 PMC8071143 33920782

[32] Jung UJ , Choi MS . Obesity and its metabolic complications: the role of adipokines and the relationship between obesity, inflammation, insulin resistance, dyslipidemia and nonalcoholic fatty liver disease. Int J Mol Sci. 2014;15:6184‐6223. doi:10.3390/ijms15046184 24733068 PMC4013623

[33] Xu T , Xu L , Meng P , et al. Angptl7 promotes insulin resistance and type 2 diabetes mellitus by multiple mechanisms including SOCS3‐mediated IRS1 degradation. FASEB J. 2020;34:13548‐13560. doi:10.1096/fj.202000246RR 32786125

[34] Horbelt T , Knebel B , Fahlbusch P , et al. The adipokine sFRP4 induces insulin resistance and lipogenesis in the liver. Biochim Biophys Acta Mol Basis Dis. 2019;1865:2671‐2684. doi:10.1016/j.bbadis.2019.07.008 31336149

[35] Ho CK , Sriram G , Dipple KM . Insulin sensitivity predictions in individuals with obesity and type II diabetes mellitus using mathematical model of the insulin signal transduction pathway. Mol Genet Metab. 2016;119:288‐292. doi:10.1016/j.ymgme.2016.09.007 27746033

[36] Pomytkin I , Costa‐Nunes JP , Kasatkin V , et al. Insulin receptor in the brain: mechanisms of activation and the role in the CNS pathology and treatment. CNS Neurosci Ther. 2018;24:763‐774. doi:10.1111/cns.12866 29691988 PMC6489906

[37] Cai W , Xue C , Sakaguchi M , et al. Insulin regulates astrocyte gliotransmission and modulates behavior. J Clin Invest. 2018;128:2914‐2926. doi:10.1172/JCI99366 29664737 PMC6025980

[38] Massarenti L , Aniol‐Nielsen C , Enevold C , Toft‐Hansen H , Nielsen CH . Influence of insulin receptor single nucleotide polymorphisms on glycaemic control and formation of anti‐insulin antibodies in diabetes mellitus. Int J Mol Sci. 2022;23:23. doi:10.3390/ijms23126481 PMC9223446 35742925

[39] Samovski D , Dhule P , Pietka T , et al. Regulation of insulin receptor pathway and glucose metabolism by CD36 signaling. Diabetes. 2018;67:1272‐1284. doi:10.2337/db17-1226 29748289 PMC6014550

[40] Mueller PL , Pritchett CE , Wiechman TN , Zharikov A , Hajnal A . Antidepressant‐like effects of insulin and IGF‐1 are mediated by IGF‐1 receptors in the brain. Brain Res Bull. 2018;143:27‐35. doi:10.1016/j.brainresbull.2018.09.017 30278200

[41] Soliman E , Essmat N , Mahmoud MF , Mahmoud AAA . Impact of some oral hypoglycemic agents on type 2 diabetes‐associated depression and reserpine‐induced depression in rats: the role of brain oxidative stress and inflammation. Naunyn Schmiedebergs Arch Pharmacol. 2020;393:1391‐1404. doi:10.1007/s00210-020-01838-w 32077986

[42] Mosili P , Mkhize BC , Ngubane P , Sibiya N , Khathi A . The dysregulation of the hypothalamic‐pituitary‐adrenal axis in diet‐induced prediabetic male Sprague Dawley rats. Nutr Metab (Lond). 2020;17:104. doi:10.1186/s12986-020-00532-1 33308255 PMC7731754

[43] Sakle N , Quraishi M , Mokale S . Ameliorative effect of quercetin and rutin via modulation of hypothala mic–pituitary–adrenal axis and regulation of fasting glucose in chroni c stress‐induced prediabetes. Phcog Mag. 2018;14:65. doi:10.4103/pm.pm_323_17

[44] Mayer SE , Lopez‐Duran NL , Sen S , Abelson JL . Chronic stress, hair cortisol and depression: a prospective and longitudinal study of medical internship. Psychoneuroendocrinology. 2018;92:57‐65. doi:10.1016/j.psyneuen.2018.03.020 29627713 PMC5924646

[45] Moica T , Gabos Grecu I , Buicu GE , et al. Increased cortisol levels in depression: a comparative study evaluating the correlation of Hypercortisolemia with prosocial coping mechanisms. Acta Medica Marisiensis. 2016;62:68‐72.

[46] Fiksdal A , Hanlin L , Kuras Y , et al. Associations between symptoms of depression and anxiety and cortisol responses to and recovery from acute stress. Psychoneuroendocrinology. 2019;102:44‐52. doi:10.1016/j.psyneuen.2018.11.035 30513499 PMC6420396

[47] Yan YX , Xiao HB , Lu YK , et al. hsa_circ_0111707 is associated with risk of stress‐related type 2 diabetes via sponging miR‐144‐3p. Front Endocrinol (Lausanne). 2021;12:790591. doi:10.3389/fendo.2021.790591 35116004 PMC8803902

[48] Boku S , Nakagawa S , Toda H , Hishimoto A . Neural basis of major depressive disorder: beyond monoamine hypothesis. Psychiatry Clin Neurosci. 2018;72:3‐12. doi:10.1111/pcn.12604 28926161

[49] Kokkinopoulou I , Diakoumi A , Moutsatsou P . Glucocorticoid receptor signaling in diabetes. Int J Mol Sci. 2021;22:22. doi:10.3390/ijms222011173 PMC8537243 34681832

[50] Opherk C , Tronche F , Kellendonk C , et al. Inactivation of the glucocorticoid receptor in hepatocytes leads to fasting hypoglycemia and ameliorates hyperglycemia in streptozotocin‐induced diabetes mellitus. Mol Endocrinol. 2004;18:1346‐1353. doi:10.1210/me.2003-0283 15031319

[51] Diz‐Chaves Y , Gil‐Lozano M , Toba L , et al. Stressing diabetes? The hidden links between insulinotropic peptides and the HPA axis. J Endocrinol. 2016;230:R77‐R94. doi:10.1530/JOE-16-0118 27325244

[52] Kinyua AW , Doan KV , Yang DJ , et al. Insulin regulates adrenal steroidogenesis by stabilizing SF‐1 activity. Sci Rep. 2018;8:5025. doi:10.1038/s41598-018-23298-2 29567944 PMC5864882

[53] Milne NT , Bucks RS , Davis WA , et al. Hippocampal atrophy, asymmetry, and cognition in type 2 diabetes mellitus. Brain Behav. 2018;8:e00741. doi:10.1002/brb3.741 29568674 PMC5853633

[54] Unver Saraydin S , Ozdenoglu Kutlu B , Saraydin D . Effects of diabetes on apoptosis and mitosis in rat hippocampus. Biotech Histochem. 2021;96:460‐467. doi:10.1080/10520295.2020.1818827 32938250

[55] Bonds JA , Shetti A , Stephen TKL , Bonini MG , Minshall RD , Lazarov O . Deficits in hippocampal neurogenesis in obesity‐dependent and ‐independent type‐2 diabetes mellitus mouse models. Sci Rep. 2020;10:16368. doi:10.1038/s41598-020-73401-9 33004912 PMC7530538

[56] Battaglia S , Orsolini S , Borgomaneri S , Barbieri R , Diciotti S , di Pellegrino G . Characterizing cardiac autonomic dynamics of fear learning in humans. Psychophysiology. 2022;59:e14122. doi:10.1111/psyp.14122 35671393 PMC9787647

[57] Battaglia S , Thayer JF . Functional interplay between central and autonomic nervous systems in human fear conditioning. Trends Neurosci. 2022;45:504‐506. doi:10.1016/j.tins.2022.04.003 35577621

[58] Barch DM , Tillman R , Kelly D , Whalen D , Gilbert K , Luby JL . Hippocampal volume and depression among young children. Psychiatry Res Neuroimaging. 2019;288:21‐28. doi:10.1016/j.pscychresns.2019.04.012 31071541 PMC6550342

[59] Chen F , Bertelsen AB , Holm IE , Nyengaard JR , Rosenberg R , Dorph‐Petersen KA . Hippocampal volume and cell number in depression, schizophrenia, and suicide subjects. Brain Res. 2020;1727:146546. doi:10.1016/j.brainres.2019.146546 31715144

[60] Vemuri P , Lesnick TG , Przybelski SA , et al. Development of a cerebrovascular magnetic resonance imaging biomarker for cognitive aging. Ann Neurol. 2018;84:705‐716. doi:10.1002/ana.25346 30264411 PMC6282853

[61] Williams OA , An Y , Beason‐Held L , et al. Vascular burden and APOE epsilon4 are associated with white matter microstructural decline in cognitively normal older adults. Neuroimage. 2019;188:572‐583. doi:10.1016/j.neuroimage.2018.12.009 30557663 PMC6601608

[62] Hou G , Lai W , Jiang W , et al. Myelin deficits in patients with recurrent major depressive disorder: an inhomogeneous magnetization transfer study. Neurosci Lett. 2021;750:135768. doi:10.1016/j.neulet.2021.135768 33636288

[63] Repple J , Konig A , de Lange SC , et al. Association between genetic risk for type 2 diabetes and structural brain connectivity in major depressive disorder. Biol Psychiatry Cogn Neurosci Neuroimaging. 2022;7:333‐340. doi:10.1016/j.bpsc.2021.02.010 33684623

[64] Zhang FF , Peng W , Sweeney JA , Jia ZY , Gong QY . Brain structure alterations in depression: psychoradiological evidence. CNS Neurosci Ther. 2018;24:994‐1003. doi:10.1111/cns.12835 29508560 PMC6489983

[65] Tanaka M , Szabo A , Spekker E , Polyak H , Toth F , Vecsei L . Mitochondrial impairment: a common motif in neuropsychiatric presentation? The link to the tryptophan‐kynurenine metabolic system. Cell. 2022;11:11. doi:10.3390/cells11162607 PMC9406499 36010683

[66] Liu K , Zhao L , Xu W , et al. Metabolic changes associated with a rat model of diabetic depression detected by ex vivo (1)H nuclear magnetic resonance spectroscopy in the prefrontal cortex, hippocampus, and hypothalamus. Neural Plast. 2018;2018:6473728. doi:10.1155/2018/6473728 29849562 PMC5911311

[67] Liu J , Liu L , Han YS , et al. The molecular mechanism underlying mitophagy‐mediated hippocampal neuron apoptosis in diabetes‐related depression. J Cell Mol Med. 2021;25:7342‐7353. doi:10.1111/jcmm.16763 34213839 PMC8335699

[68] Wang X , Hu WH , Zhang K , et al. Acute fornix deep brain stimulation improves hippocampal glucose metabolism in aged mice. Chin Med J (Engl). 2018;131:594‐599. doi:10.4103/0366-6999.226067 29483395 PMC5850677

[69] Guan J , Ding Y , Rong Y , et al. Early life stress increases brain glutamate and induces neurobehavioral manifestations in rats. ACS Chem Nerosci. 2020;11:4169‐4178. doi:10.1021/acschemneuro.0c00454 33179901

[70] Li DX , Wang CN , Wang Y , et al. NLRP3 inflammasome‐dependent pyroptosis and apoptosis in hippocampus neurons mediates depressive‐like behavior in diabetic mice. Behav Brain Res. 2020;391:112684. doi:10.1016/j.bbr.2020.112684 32454054

[71] Harrison NJ , Connolly E , Gascon Gubieda A , et al. Regenerative neurogenic response from glia requires insulin‐driven neuron‐glia communication. Elife. 2021;10:10. doi:10.7554/eLife.58756 PMC7880684 33527895

[72] Otsuki L , Brand AH . Cell cycle heterogeneity directs the timing of neural stem cell activation from quiescence. Science. 2018;360:99‐102. doi:10.1126/science.aan8795 29622651 PMC6538531

[73] Spinelli M , Natale F , Rinaudo M , et al. Neural stem cell‐derived exosomes revert HFD‐dependent memory impairment via CREB‐BDNF signalling. Int J Mol Sci. 2020;21:21. doi:10.3390/ijms21238994 PMC7729830 33256199

[74] Gil‐Ranedo J , Gonzaga E , Jaworek KJ , Berger C , Bossing T , Barros CS . STRIPAK members orchestrate hippo and insulin receptor signaling to promote neural stem cell reactivation. Cell Rep. 2019;27(10):2921‐2933.e5. doi:10.1016/j.celrep.2019.05.023 31167138 PMC6581792

[75] Huang J , Wang H . Hsp83/Hsp90 physically associates with insulin receptor to promote neural stem cell reactivation. Stem Cell Reports. 2018;11:883‐896. doi:10.1016/j.stemcr.2018.08.014 30245208 PMC6178561

[76] Prabu P , Poongothai S , Shanthirani CS , Anjana RM , Mohan V , Balasubramanyam M . Altered circulatory levels of miR‐128, BDNF, cortisol and shortened telomeres in patients with type 2 diabetes and depression. Acta Diabetol. 2020;57:799‐807. doi:10.1007/s00592-020-01486-9 32025863

[77] Bi C , Fu Y , Li B . Brain‐derived neurotrophic factor alleviates diabetes mellitus‐accelerated atherosclerosis by promoting M2 polarization of macrophages through repressing the STAT3 pathway. Cell Signal. 2020;70:109569. doi:10.1016/j.cellsig.2020.109569 32061924

[78] Wosiski‐Kuhn M , Bota M , Snider CA , et al. Hippocampal brain‐derived neurotrophic factor determines recruitment of anatomically connected networks after stress in diabetic mice. Hippocampus. 2018;28:900‐912. doi:10.1002/hipo.23018 30098276 PMC6544163

[79] Lorenzetti V , Costafreda SG , Rimmer RM , Rasenick MM , Marangell LB , Fu CHY . Brain‐derived neurotrophic factor association with amygdala response in major depressive disorder. J Affect Disord. 2020;267:103‐106. doi:10.1016/j.jad.2020.01.159 32063560 PMC8020847

[80] Moroianu LA , Cecilia C , Ardeleanu V , et al. Clinical study of serum serotonin as a screening marker for anxiety and depression in patients with type 2 diabetes. Medicina (Kaunas). 2022;58:652. doi:10.3390/medicina58050652 35630069 PMC9146121

[81] Zhuang X , Xu H , Fang Z , Xu C , Xue C , Hong X . Platelet serotonin and serotonin transporter as peripheral surrogates in depression and anxiety patients. Eur J Pharmacol. 2018;834:213‐220. doi:10.1016/j.ejphar.2018.07.033 30031795

[82] Aleksovski B , Novotni A , Vujovic V , et al. Evaluation of peripheral serotonin content and alpha2‐adrenergic receptor function as potential markers for life‐long recurrent depressive disorder by using methodological improvements. Int J Psychiatry Clin Pract. 2018;22:215‐224. doi:10.1080/13651501.2017.1411516 29216784

[83] Li H , Liu Y , Gao X , et al. Neuroplastin 65 modulates anxiety‐ and depression‐like behavior likely through adult hippocampal neurogenesis and central 5‐HT activity. FEBS J. 2019;286:3401‐3415. doi:10.1111/febs.14865 31034748

[84] Perez‐Palomar B , Mollinedo‐Gajate I , Berrocoso E , Meana JJ , Ortega JE . Serotonin 5‐HT3 receptor antagonism potentiates the antidepressant activity of citalopram. Neuropharmacology. 2018;133:491‐502. doi:10.1016/j.neuropharm.2018.02.020 29477299

[85] Wang Q , Dong X , Hu T , et al. Constitutive activity of serotonin receptor 6 regulates human cerebral organoids formation and depression‐like behaviors. Stem Cell Reports. 2021;16:75‐88. doi:10.1016/j.stemcr.2020.11.015 33357407 PMC7815944

[86] Sakimura K , Maekawa T , Sasagawa K , Ishii Y , Kume SI , Ohta T . Depression‐related behavioural and neuroendocrine changes in the Spontaneously Diabetic Torii (SDT) fatty rat, an animal model of type 2 diabetes mellitus. Clin Exp Pharmacol Physiol. 2018;45:927‐933. doi:10.1111/1440-1681.12965 29757463

[87] Parashar A , Mehta V , Malairaman U . Type 2 diabetes mellitus is associated with social recognition memory deficit and altered dopaminergic neurotransmission in the amygdala. Ann Neurosci. 2018;24:212‐220. doi:10.1159/000479637 29849445 PMC5969354

[88] Aslanoglou D , Bertera S , Sanchez‐Soto M , et al. Dopamine regulates pancreatic glucagon and insulin secretion via adrenergic and dopaminergic receptors. Transl Psychiatry. 2021;11:59. doi:10.1038/s41398-020-01171-z 33589583 PMC7884786

[89] Bini J , Sanchez‐Rangel E , Gallezot JD , et al. PET imaging of pancreatic dopamine D2 and D3 receptor density with (11)C‐(+)‐PHNO in type 1 diabetes. J Nucl Med. 2020;61:570‐576. doi:10.2967/jnumed.119.234013 31601695 PMC7198375

[90] Meng F , Guo Z , Hu Y , et al. CD73‐derived adenosine controls inflammation and neurodegeneration by modulating dopamine signalling. Brain. 2019;142:700‐718. doi:10.1093/brain/awy351 30689733

[91] Shimojo G , Joseph B , Shah R , Consolim‐Colombo FM , De Angelis K , Ulloa L . Exercise activates vagal induction of dopamine and attenuates systemic inflammation. Brain Behav Immun. 2019;75:181‐191. doi:10.1016/j.bbi.2018.10.005 30394312 PMC6334665

[92] Feketeova E , Li Z , Joseph B , Shah R , Spolarics Z , Ulloa L . Dopaminergic control of inflammation and Glycemia in sepsis and diabetes. Front Immunol. 2018;9:943. doi:10.3389/fimmu.2018.00943 29780390 PMC5945822

[93] Bittencourt A , Brum PO , Ribeiro CT , et al. High fat diet‐induced obesity causes a reduction in brain tyrosine hydroxylase levels and non‐motor features in rats through metabolic dysfunction, neuroinflammation and oxidative stress. Nutr Neurosci. 2022;25:1026‐1040. doi:10.1080/1028415X.2020.1831261 33078695

[94] Barry RL , Byun NE , Williams JM , et al. Brief exposure to obesogenic diet disrupts brain dopamine networks. PloS One. 2018;13:e0191299. doi:10.1371/journal.pone.0191299 29698491 PMC5919534

[95] Gao N , Tiliwaerde M , Zheng W , Xiong J , Li X , Jin Z . Neuropharmacological and antidepressant‐like effects of ZY‐1408: a novel serotonin/norepinephrine reuptake inhibitor and serotonin receptor 2C antagonist. Neuropharmacology. 2021;182:108376. doi:10.1016/j.neuropharm.2020.108376 33122031

[96] Cao S , Fisher DW , Rodriguez G , Yu T , Dong H . Comparisons of neuroinflammation, microglial activation, and degeneration of the locus coeruleus‐norepinephrine system in APP/PS1 and aging mice. J Neuroinflammation. 2021;18:10. doi:10.1186/s12974-020-02054-2 33407625 PMC7789762

[97] Sugama S , Takenouchi T , Hashimoto M , Ohata H , Takenaka Y , Kakinuma Y . Stress‐induced microglial activation occurs through beta‐adrenergic receptor: noradrenaline as a key neurotransmitter in microglial activation. J Neuroinflammation. 2019;16:266. doi:10.1186/s12974-019-1632-z 31847911 PMC6916186

[98] Stefanovic B , Spasojevic N , Jovanovic P , Dronjak S . Melatonin treatment affects changes in adrenal gene expression of catecholamine biosynthesizing enzymes and norepinephrine transporter in the rat model of chronic‐stress‐induced depression. Can J Physiol Pharmacol. 2019;97:685‐690. doi:10.1139/cjpp-2018-0612 30773040

[99] Meng L , Bai X , Zheng Y , Chen D , Zheng Y . Altered expression of norepinephrine transporter participate in hypertension and depression through regulated TNF‐alpha and IL‐6. Clin Exp Hypertens. 2020;42:181‐189. doi:10.1080/10641963.2019.1601205 30957546

[100] Davis MA , Camacho LE , Anderson MJ , et al. Chronically elevated norepinephrine concentrations lower glucose uptake in fetal sheep. Am J Physiol Regul Integr Comp Physiol. 2020;319:R255‐R263. doi:10.1152/ajpregu.00365.2019 32667834 PMC7509250

[101] Yang QQ , Shao D , Li J , Yang CL , Fan MH , Cao FL . Positive association between serum levels of high‐sensitivity C‐reactive protein and depression/anxiety in female, but not male, patients with type 2 diabetes mellitus. Biol Res Nurs. 2020;22:178‐187. doi:10.1177/1099800419894641 31867989

[102] Huang Y , Su Y , Chen H , Liu H , Hu J . Serum levels of CRP are associated with depression in a middle‐aged and elderly population with diabetes mellitus: a diabetes mellitus‐stratified analysis in a population‐based study. J Affect Disord. 2021;281:351‐357. doi:10.1016/j.jad.2020.12.028 33348178

[103] Shafiee M , Ahmadnezhad M , Tayefi M , et al. Depression and anxiety symptoms are associated with prooxidant‐antioxidant balance: a population‐based study. J Affect Disord. 2018;238:491‐498. doi:10.1016/j.jad.2018.05.079 29935471

[104] Bhatt S , Nagappa AN , Patil CR . Role of oxidative stress in depression. Drug Discov Today. 2020;25:1270‐1276. doi:10.1016/j.drudis.2020.05.001 32404275

[105] Islam MR , Ali S , Karmoker JR , et al. Evaluation of serum amino acids and non‐enzymatic antioxidants in drug‐naive first‐episode major depressive disorder. BMC Psychiatry. 2020;20:333. doi:10.1186/s12888-020-02738-2 32580709 PMC7315550

[106] Lu YR , Zhang Y , Rao YB , et al. The changes in, and relationship between, plasma nitric oxide and corticotropin‐releasing hormone in patients with major depressive disorder. Clin Exp Pharmacol Physiol. 2018;45:10‐15. doi:10.1111/1440-1681.12826 28755509 PMC6084347

[107] Ghasemi M . Nitric oxide: antidepressant mechanisms and inflammation. Adv Pharmacol. 2019;86:121‐152. doi:10.1016/bs.apha.2019.04.004 31378250

[108] McNeill RV , Kehrwald C , Brum M , et al. Uncovering associations between mental illness diagnosis, nitric oxide synthase gene variation, and peripheral nitric oxide concentration. Brain Behav Immun. 2022;101:275‐283. doi:10.1016/j.bbi.2022.01.006 35041938

[109] Karabulut D , Sonmez MF . Effects of diabetes on nitric oxide synthase in rat uterus. Biotech Histochem. 2021;96:331‐338. doi:10.1080/10520295.2020.1788161 32654526

[110] Melin EO , Dereke J , Hillman M . Low levels of soluble TWEAK, indicating on‐going inflammation, were associated with depression in type 1 diabetes: a cross‐sectional study. BMC Psychiatry. 2020;20:574. doi:10.1186/s12888-020-02977-3 33261587 PMC7709277

[111] Li ZR , Han YS , Liu Z , et al. GR/NF‐kappaB signaling pathway regulates hippocampal inflammatory responses in diabetic rats with chronic unpredictable mild stress. Eur J Pharmacol. 2021;895:173861. doi:10.1016/j.ejphar.2021.173861 33465356

[112] Cheng Y , Desse S , Martinez A , Worthen RJ , Jope RS , Beurel E . TNFalpha disrupts blood brain barrier integrity to maintain prolonged depressive‐like behavior in mice. Brain Behav Immun. 2018;69:556‐567. doi:10.1016/j.bbi.2018.02.003 29452218 PMC5963697

[113] Zhou R , Wang F , Zhao G , et al. Effects of tumor necrosis factor‐alpha polymorphism on the brain structural changes of the patients with major depressive disorder. Transl Psychiatry. 2018;8:217. doi:10.1038/s41398-018-0256-x 30310056 PMC6181976

[114] Trapali M , Houhoula D , Batrinou A , et al. Association of TNF‐alpha 308G/a and LEPR Gln223Arg polymorphisms with the risk of type 2 diabetes mellitus. Genes (Basel). 2021;13:13. doi:10.3390/genes13010059 35052401 PMC8796026

[115] Lampropoulou IT , Stangou M , Sarafidis P , et al. TNF‐alpha pathway and T‐cell immunity are activated early during the development of diabetic nephropathy in type II diabetes mellitus. Clin Immunol. 2020;215:108423. doi:10.1016/j.clim.2020.108423 32304735

[116] Ghosh R , Kumar PK , Mitra P , Purohit P , Nebhinani N , Sharma P . Circulating T helper 17 and IFN‐gamma positive Th17 cells in major depressive disorder. Behav Brain Res. 2020;394:112811. doi:10.1016/j.bbr.2020.112811 32702351

[117] Schiweck C , Valles‐Colomer M , Arolt V , et al. Depression and suicidality: a link to premature T helper cell aging and increased Th17 cells. Brain Behav Immun. 2020;87:603‐609. doi:10.1016/j.bbi.2020.02.005 32061905

[118] Medina‐Rodriguez EM , Madorma D , O'Connor G , et al. Identification of a signaling mechanism by which the microbiome regulates Th17 cell‐mediated depressive‐like behaviors in mice. Am J Psychiatry. 2020;177:974‐990. doi:10.1176/appi.ajp.2020.19090960 32731813 PMC7647050

[119] Nicholas DA , Proctor EA , Agrawal M , et al. Fatty acid metabolites combine with reduced beta oxidation to activate Th17 inflammation in human type 2 diabetes. Cell Metab. 2019;30(3):447‐461.e5. doi:10.1016/j.cmet.2019.07.004 31378464 PMC8506657

[120] Costa FRC , Leite JA , Rassi DM , et al. NLRP1 acts as a negative regulator of Th17 cell programming in mice and humans with autoimmune diabetes. Cell Rep. 2021;35:109176. doi:10.1016/j.celrep.2021.109176 34038731

[121] Wang Y , Li W , Zhou J , et al. Autoantibody‐positivity in lean type II diabetes patients was associated with elevated Th17‐like CD4(+)CXCR5(+) T cell responses. Mol Immunol. 2019;112:305‐311. doi:10.1016/j.molimm.2019.06.010 31229843

[122] Rutman AK , Negi S , Gasparrini M , Hasilo CP , Tchervenkov J , Paraskevas S . Immune response to extracellular vesicles from human islets of Langerhans in patients with type 1 diabetes. Endocrinology. 2018;159:3834‐3847. doi:10.1210/en.2018-00649 30307543

[123] Kahleova H , Rembert E , Alwarith J , et al. Effects of a low‐fat vegan diet on gut microbiota in overweight individuals and relationships with body weight, body composition, and insulin sensitivity. A randomized clinical trial. Nutrients. 2020;12:12. doi:10.3390/nu12102917 PMC7598634 32987642

[124] Khalyfa A , Ericsson A , Qiao Z , Almendros I , Farre R , Gozal D . Circulating exosomes and gut microbiome induced insulin resistance in mice exposed to intermittent hypoxia: effects of physical activity. EBioMedicine. 2021;64:103208. doi:10.1016/j.ebiom.2021.103208 33485839 PMC7910674

[125] Heianza Y , Sun D , Li X , et al. Gut microbiota metabolites, amino acid metabolites and improvements in insulin sensitivity and glucose metabolism: the POUNDS lost trial. Gut. 2019;68:263‐270. doi:10.1136/gutjnl-2018-316155 29860242 PMC6275143

[126] Jiao N , Baker SS , Nugent CA , et al. Gut microbiome may contribute to insulin resistance and systemic inflammation in obese rodents: a meta‐analysis. Physiol Genomics. 2018;50:244‐254. doi:10.1152/physiolgenomics.00114.2017 29373083

[127] Wang H , Lu Y , Yan Y , et al. Promising treatment for type 2 diabetes: fecal microbiota transplantation reverses insulin resistance and impaired islets. Front Cell Infect Microbiol. 2019;9:455. doi:10.3389/fcimb.2019.00455 32010641 PMC6979041

[128] Sun L , Ma L , Zhang H , et al. Fto deficiency reduces anxiety‐and depression‐like behaviors in mice via alterations in gut microbiota. Theranostics. 2019;9:721‐733. doi:10.7150/thno.31562 30809304 PMC6376469

[129] Chahwan B , Kwan S , Isik A , van Hemert S , Burke C , Roberts L . Gut feelings: a randomised, triple‐blind, placebo‐controlled trial of probiotics for depressive symptoms. J Affect Disord. 2019;253:317‐326. doi:10.1016/j.jad.2019.04.097 31078831

[130] Liu RT , Walsh RFL , Sheehan AE . Prebiotics and probiotics for depression and anxiety: a systematic review and meta‐analysis of controlled clinical trials. Neurosci Biobehav Rev. 2019;102:13‐23. doi:10.1016/j.neubiorev.2019.03.023 31004628 PMC6584030

[131] Reininghaus EZ , Platzer M , Kohlhammer‐Dohr A , et al. PROVIT: supplementary probiotic treatment and vitamin B7 in depression‐a randomized controlled trial. Nutrients. 2020;12:12. doi:10.3390/nu12113422 PMC7695208 33171595

[132] Ning F , Zhang D , Xue B , et al. Synergistic effects of depression and obesity on type 2 diabetes incidence in Chinese adults. J Diabetes. 2020;12:142‐150. doi:10.1111/1753-0407.12968 31287240

[133] Huang B , Huang Z , Tan J , et al. The mediating and interacting role of physical activity and sedentary behavior between diabetes and depression in people with obesity in United States. J Diabetes Complications. 2021;35:107764. doi:10.1016/j.jdiacomp.2020.107764 33616042

[134] Senkus KE , Crowe‐White KM , Bolland AC , Locher JL , Ard JD . Changes in adiponectin:leptin ratio among older adults with obesity following a 12‐month exercise and diet intervention. Nutr Diabetes. 2022;12:30. doi:10.1038/s41387-022-00207-1 35654771 PMC9163185

[135] Khoramipour K , Chamari K , Hekmatikar AA , et al. Adiponectin: structure, physiological functions, role in diseases, and effects of nutrition. Nutrients. 2021;13:13. doi:10.3390/nu13041180 PMC8066826 33918360

[136] Cao B , Chen Y , Brietzke E , et al. Leptin and adiponectin levels in major depressive disorder: a systematic review and meta‐analysis. J Affect Disord. 2018;238:101‐110. doi:10.1016/j.jad.2018.05.008 29870819

[137] Cernea S , Both E , Hutanu A , Sular FL , Roiban AL . Correlations of serum leptin and leptin resistance with depression and anxiety in patients with type 2 diabetes. Psychiatry Clin Neurosci. 2019;73:745‐753. doi:10.1111/pcn.12922 31404477

[138] Baye E , Ukropec J , de Courten MPJ , et al. Carnosine supplementation improves serum resistin concentrations in overweight or obese otherwise healthy adults: a pilot randomized trial. Nutrients. 2018;10:10. doi:10.3390/nu10091258 PMC6165206 30205427

[139] Rahman S , Shanta AA , Daria S , et al. Increased serum resistin but not G‐CSF levels are associated in the pathophysiology of major depressive disorder: findings from a case‐control study. PloS One. 2022;17:e0264404. doi:10.1371/journal.pone.0264404 35213631 PMC8880862

[140] Derosa G , Catena G , Gaudio G , D'Angelo A , Maffioli P . Adipose tissue dysfunction and metabolic disorders: is it possible to predict who will develop type 2 diabetes mellitus? Role of markErs in the progreSsion of dIabeteS in obese paTIeNts (the RESISTIN trial). Cytokine. 2020;127:154947. doi:10.1016/j.cyto.2019.154947 31811995

[141] Dias GD , Cartolano FC , Freitas MCP , et al. Adiponectin predicts the antioxidant capacity and size of high‐density lipoprotein (HDL) in individuals with diabetes mellitus. J Diabetes Complications. 2021;35:107856. doi:10.1016/j.jdiacomp.2021.107856 33627254

[142] Hashimoto H , Yamamoto M , Sugiura E , et al. Adiponectin deficiency‐induced diabetes increases TNFalpha and FFA via downregulation of PPARalpha. J Vet Med Sci. 2018;80:662‐666. doi:10.1292/jvms.17-0641 29445073 PMC5938197

[143] Formolo DA , Lee TH , Yau SY . Increasing adiponergic system activity as a potential treatment for depressive disorders. Mol Neurobiol. 2019;56:7966‐7976. doi:10.1007/s12035-019-01644-3 31140056 PMC6834732

[144] You J , Sun L , Wang J , et al. Role of adiponectin‐notch pathway in cognitive dysfunction associated with depression and in the therapeutic effect of physical exercise. Aging Cell. 2021;20:e13387. doi:10.1111/acel.13387 34053165 PMC8208781

[145] Qin X , Wang W , Wu H , et al. PPARgamma‐mediated microglial activation phenotype is involved in depressive‐like behaviors and neuroinflammation in stressed C57BL/6J and Ob/Ob mice. Psychoneuroendocrinology. 2020;117:104674. doi:10.1016/j.psyneuen.2020.104674 32422516

[146] de Carvalho‐Ferreira JP , Masquio DC , da Silveira Campos RM , et al. Is there a role for leptin in the reduction of depression symptoms during weight loss therapy in obese adolescent girls and boys? Peptides. 2015;65:20‐28. doi:10.1016/j.peptides.2014.11.010 25629253

[147] Bidulescu A , Dinh PC Jr , Sarwary S , et al. Associations of leptin and adiponectin with incident type 2 diabetes and interactions among African Americans: the Jackson heart study. BMC Endocr Disord. 2020;20:31. doi:10.1186/s12902-020-0511-z 32131811 PMC7057597

[148] Moon HS , Matarese G , Brennan AM , et al. Efficacy of metreleptin in obese patients with type 2 diabetes: cellular and molecular pathways underlying leptin tolerance. Diabetes. 2011;60:1647‐1656. doi:10.2337/db10-1791 21617185 PMC3114380

[149] Rebolledo‐Solleiro D , Roldan‐Roldan G , Diaz D , et al. Increased anxiety‐like behavior is associated with the metabolic syndrome in non‐stressed rats. PloS One. 2017;12:e0176554. doi:10.1371/journal.pone.0176554 28463967 PMC5413000

[150] Kaplon‐Cieslicka A , Tyminska A , Rosiak M , et al. Resistin is a prognostic factor for death in type 2 diabetes. Diabetes Metab Res Rev. 2019;35:e3098. doi:10.1002/dmrr.3098 30447052

[151] Tsiotra PC , Boutati E , Dimitriadis G , Raptis SA . High insulin and leptin increase resistin and inflammatory cytokine production from human mononuclear cells. Biomed Res Int. 2013;2013:487081. doi:10.1155/2013/487081 23484124 PMC3591160

[152] Vancampfort D , Correll CU , Galling B , et al. Diabetes mellitus in people with schizophrenia, bipolar disorder and major depressive disorder: a systematic review and large scale meta‐analysis. World Psychiatry. 2016;15:166‐174. doi:10.1002/wps.20309 27265707 PMC4911762

[153] Barton BB , Segger F , Fischer K , Obermeier M , Musil R . Update on weight‐gain caused by antipsychotics: a systematic review and meta‐analysis. Expert Opin Drug Saf. 2020;19:295‐314. doi:10.1080/14740338.2020.1713091 31952459

[154] Kishi T , Ikuta T , Matsuda Y , Iwata N . Quetiapine extended‐release vs olanzapine for Japanese patients with bipolar depression: a Bayesian analysis. Neuropsychopharmacol Rep. 2019;39:256‐259. doi:10.1002/npr2.12070 31283865 PMC7292317

[155] Croatto G , Vancampfort D , Miola A , et al. The impact of pharmacological and non‐pharmacological interventions on physical health outcomes in people with mood disorders across the lifespan: An umbrella review of the evidence from randomised controlled trials. Mol Psychiatry. 2023;28:369‐390. doi:10.1038/s41380-022-01770-w 36138129 PMC9493151

[156] Lappin JM , Wijaya M , Watkins A , et al. Cardio‐metabolic risk and its management in a cohort of clozapine‐treated outpatients. Schizophr Res. 2018;199:367‐373. doi:10.1016/j.schres.2018.02.035 29486959

[157] Quek YF , See YM , Yee JY , et al. Metabolic syndrome and cardiovascular risk between clozapine and non‐clozapine antipsychotic users with schizophrenia. Asian J Psychiatr. 2022;74:103192. doi:10.1016/j.ajp.2022.103192 35751958

[158] Tsai HP , Hou PH , Mao FC , et al. Risperidone exacerbates glucose intolerance, nonalcoholic fatty liver disease, and renal impairment in obese mice. Int J Mol Sci. 2021;22:22. doi:10.3390/ijms22010409 PMC7795724 33401717

[159] Findling RL , Pathak S , Earley WR , Liu S , DelBello MP . Efficacy and safety of extended‐release quetiapine fumarate in youth with bipolar depression: an 8 week, double‐blind, placebo‐controlled trial. J Child Adolesc Psychopharmacol. 2014;24:325‐335. doi:10.1089/cap.2013.0105 24956042 PMC4137347

[160] Srinivas S , Parvataneni T , Makani R , Patel RS . Efficacy and safety of quetiapine for pediatric bipolar depression: a systematic review of randomized clinical trials. Cureus. 2020;12:e8407. doi:10.7759/cureus.8407 32637286 PMC7331915

[161] Chiu YJ , Tu HH , Kung ML , Wu HJ , Chen YW . Fluoxetine ameliorates high‐fat diet‐induced metabolic abnormalities partially via reduced adipose triglyceride lipase‐mediated adipocyte lipolysis. Biomed Pharmacother. 2021;141:111848. doi:10.1016/j.biopha.2021.111848 34198047

[162] Chen HM , Yang YH , Chen KJ , et al. Antidepressants reduced risk of mortality in patients with diabetes mellitus: a population‐based cohort study in Taiwan. J Clin Endocrinol Metab. 2019;104:4619‐4625. doi:10.1210/jc.2018-02362 31265070

[163] Avrahamy H , Shoval G , Hoshen M , et al. Association between adherence to SSRI treatment and mortality among individuals with metabolic syndrome components. Pharmacopsychiatry. 2021;54:232‐239. doi:10.1055/a-1425-7246 33853176

[164] Nestsiarovich A , Kerner B , Mazurie AJ , et al. Diabetes mellitus risk for 102 drugs and drug combinations used in patients with bipolar disorder. Psychoneuroendocrinology. 2020;112:104511. doi:10.1016/j.psyneuen.2019.104511 31744781

[165] Visco DB , Manhaes‐de‐Castro R , Chaves WF , et al. Selective serotonin reuptake inhibitors affect structure, function and metabolism of skeletal muscle: a systematic review. Pharmacol Res. 2018;136:194‐204. doi:10.1016/j.phrs.2018.09.004 30196103

[166] Rasmussen NH , Dal J , den Bergh JV , de Vries F , Jensen MH , Vestergaard P . Increased risk of falls, fall‐related injuries and fractures in people with type 1 and type 2 diabetes–a nationwide cohort study. Curr Drug Saf. 2021;16:52‐61. doi:10.2174/1574886315666200908110058 32900349

[167] Matta M , Pavy‐Le Traon A , Perez‐Lloret S , et al. Predictors of cardiovascular autonomic neuropathy onset and progression in a cohort of type 1 diabetic patients. J Diabetes Res. 2018;2018:5601351. doi:10.1155/2018/5601351 29693021 PMC5859848

[168] Yu H , Zhang Y , Xing C , et al. Venlafaxine Caffeic acid salt: synthesis, structural characterization, and hypoglycemic effect analysis. ACS Omega. 2021;6:13895‐13903. doi:10.1021/acsomega.1c01581 34095681 PMC8173613

[169] Gagnon J , Lussier MT , MacGibbon B , Daskalopoulou SS , Bartlett G . The impact of antidepressant therapy on glycemic control in Canadian primary care patients with diabetes mellitus. Front Nutr. 2018;5:47. doi:10.3389/fnut.2018.00047 29946546 PMC6005871

[170] Nguyen TTH , Roussin A , Rousseau V , Montastruc JL , Montastruc F . Role of serotonin transporter in antidepressant‐induced diabetes mellitus: a Pharmacoepidemiological‐Pharmacodynamic study in VigiBase((R)). Drug Saf. 2018;41:1087‐1096. doi:10.1007/s40264-018-0693-8 29956218

[171] Gong N , Zhang Y , Wang Y , et al. Versatile salts as a strategy to modify the biopharmaceutical properties of venlafaxine and a potential hypoglycemic effect study. Cryst Growth des. 2020;20:3131‐3139. doi:10.1021/acs.cgd.0c00007

[172] Alvarez‐Mon MA , Garcia‐Montero C , Fraile‐Martinez O , et al. Current opinions about the use of duloxetine: results from a survey aimed at psychiatrists. Brain Sci. 2023;13:13. doi:10.3390/brainsci13020333 PMC9953910 36831876

[173] Khoza S , Barner JC , Bohman TM , Rascati K , Lawson K , Wilson JP . Use of antidepressant agents and the risk of type 2 diabetes. Eur J Clin Pharmacol. 2012;68:1295‐1302. doi:10.1007/s00228-011-1168-3 22120432

[174] Burcu M , Zito JM , Safer DJ , et al. Association of antidepressant medications with incident type 2 diabetes among Medicaid‐insured youths. JAMA Pediatr. 2017;171:1200‐1207. doi:10.1001/jamapediatrics.2017.2896 29049533 PMC6583651

[175] Burcu M , Zito JM , Safer DJ , et al. Concomitant use of atypical antipsychotics with other psychotropic medication classes and the risk of type 2 diabetes mellitus. J Am Acad Child Adolesc Psychiatry. 2017;56:642‐651. doi:10.1016/j.jaac.2017.04.004 28735693

[176] Reis ACD , Cunha MV , Bianchin MA , Freitas MTR , Castiglioni L . Comparison of quality of life and functionality in type 2 diabetics with and without insulin. Rev Assoc Med Bras. 1992;2019(65):1464‐1469. doi:10.1590/1806-9282.65.12.1464 31994627

[177] Bai X , Liu Z , Li Z , Yan D . The association between insulin therapy and depression in patients with type 2 diabetes mellitus: a meta‐analysis. BMJ Open. 2018;8:e020062. doi:10.1136/bmjopen-2017-020062 PMC6278799 30498035

[178] Ng QX , Venkatanarayanan N , Ho CY . Clinical use of hypericum perforatum (St John's wort) in depression: a meta‐analysis. J Affect Disord. 2017;210:211‐221. doi:10.1016/j.jad.2016.12.048 28064110

[179] Hagger V , Hendrieckx C , Sturt J , Skinner TC , Speight J . Diabetes distress among adolescents with type 1 diabetes: a systematic review. Curr Diab Rep. 2016;16:9. doi:10.1007/s11892-015-0694-2 26748793

[180] Dalal MR , Kazemi M , Ye F , Xie L . Hypoglycemia after initiation of basal insulin in patients with type 2 diabetes in the United States: implications for treatment discontinuation and healthcare costs and utilization. Adv Ther. 2017;34:2083‐2092. doi:10.1007/s12325-017-0592-x 28779282 PMC5599444

[181] Cakici N , Bot M , Lamers F , et al. Increased serum levels of leptin and insulin in both schizophrenia and major depressive disorder: a cross‐disorder proteomics analysis. Eur Neuropsychopharmacol. 2019;29:835‐846. doi:10.1016/j.euroneuro.2019.05.010 31230885

[182] Anjom‐Shoae J , Hassanzadeh Keshteli A , Afshar H , Esmaillzadeh A , Adibi P . Association between dietary insulin index and load and psychological disorders. Br J Nutr. 2020;123:161‐171. doi:10.1017/S0007114519002575 31601278

[183] Khan WA , Malik A , Khan MWA . Depression linked to higher antibodies production against estrogenized insulin in type 1 diabetes. Int Immunopharmacol. 2020;86:106712. doi:10.1016/j.intimp.2020.106712 32585610

[184] Calkins‐Smith AK , Marker AM , Clements MA , Patton SR . Hope and mealtime insulin boluses are associated with depressive symptoms and glycemic control in youth with type 1 diabetes mellitus. Pediatr Diabetes. 2018;19:1309‐1314. doi:10.1111/pedi.12695 29797445 PMC6175638

[185] Zhao F , Siu JJ , Huang W , Askwith C , Cao L . Insulin modulates excitatory synaptic transmission and synaptic plasticity in the mouse hippocampus. Neuroscience. 2019;411:237‐254. doi:10.1016/j.neuroscience.2019.05.033 31146008 PMC6612444

[186] Kessing LV , Rytgaard HC , Ekstrom CT , Knop FK , Berk M , Gerds TA . Antidiabetes agents and incident depression: a nationwide population‐based study. Diabetes Care. 2020;43:3050‐3060. doi:10.2337/dc20-1561 32978179

[187] Chen F , Wei G , Wang Y , et al. Risk factors for depression in elderly diabetic patients and the effect of metformin on the condition. BMC Public Health. 2019;19:1063. doi:10.1186/s12889-019-7392-y 31391021 PMC6686369

[188] Chin SO , Ha IG , Rhee SY , et al. Clinical characteristics and prevalence of comorbidities according to metformin use in Korean patients with type 2 diabetes. Int J Endocrinol. 2020;2020:9879517. doi:10.1155/2020/9879517 32774367 PMC7396103

[189] Wium‐Andersen IK , Osler M , Jorgensen MB , Rungby J , Wium‐Andersen MK . Diabetes, antidiabetic medications and risk of depression–a population‐based cohort and nested case‐control study. Psychoneuroendocrinology. 2022;140:105715. doi:10.1016/j.psyneuen.2022.105715 35338947

[190] Chen J , Zhou T , Guo AM , et al. Metformin ameliorates lipopolysaccharide‐induced depressive‐like behaviors and abnormal glutamatergic transmission. Biology (Basel). 2020;9:9. doi:10.3390/biology9110359 PMC7692296 33114529

[191] Zemdegs J , Martin H , Pintana H , et al. Metformin promotes anxiolytic and antidepressant‐like responses in insulin‐resistant mice by decreasing circulating branched‐chain amino acids. J Neurosci. 2019;39:5935‐5948. doi:10.1523/JNEUROSCI.2904-18.2019 31160539 PMC6650994

[192] Yang S , Chen X , Xu Y , Hao Y , Meng X . Effects of metformin on lipopolysaccharide‐induced depressive‐like behavior in mice and its mechanisms. Neuroreport. 2020;31:305‐310. doi:10.1097/WNR.0000000000001401 31977586

[193] Fang W , Zhang J , Hong L , et al. Metformin ameliorates stress‐induced depression‐like behaviors via enhancing the expression of BDNF by activating AMPK/CREB‐mediated histone acetylation. J Affect Disord. 2020;260:302‐313. doi:10.1016/j.jad.2019.09.013 31521867

[194] Lam YY , Tsai SF , Chen PC , Kuo YM , Chen YW . Pioglitazone rescues high‐fat diet‐induced depression‐like phenotypes and hippocampal astrocytic deficits in mice. Biomed Pharmacother. 2021;140:111734. doi:10.1016/j.biopha.2021.111734 34022606

[195] Zhao Q , Wu X , Yan S , et al. The antidepressant‐like effects of pioglitazone in a chronic mild stress mouse model are associated with PPARgamma‐mediated alteration of microglial activation phenotypes. J Neuroinflammation. 2016;13:259. doi:10.1186/s12974-016-0728-y 27716270 PMC5051050

[196] Li J , Xu B , Chen Z , et al. PI3K/AKT/JNK/p38 signalling pathway‐mediated neural apoptosis in the prefrontal cortex of mice is involved in the antidepressant‐like effect of pioglitazone. Clin Exp Pharmacol Physiol. 2018;45:525‐535. doi:10.1111/1440-1681.12918 29359338

[197] Beheshti F , Hosseini M , Hashemzehi M , Soukhtanloo M , Asghari A . The effects of PPAR‐γ agonist pioglitazone on anxiety and depression‐like behaviors in lipopolysaccharide injected rats. Toxin Reviews. 2019;40:1223‐1232. doi:10.1080/15569543.2019.1673425

[198] Liao L , Zhang XD , Li J , et al. Pioglitazone attenuates lipopolysaccharide‐induced depression‐like behaviors, modulates NF‐kappaB/IL‐6/STAT3, CREB/BDNF pathways and central serotonergic neurotransmission in mice. Int Immunopharmacol. 2017;49:178‐186. doi:10.1016/j.intimp.2017.05.036 28595081

[199] Aftab A , Kemp DE , Ganocy SJ , et al. Double‐blind, placebo‐controlled trial of pioglitazone for bipolar depression. J Affect Disord. 2019;245:957‐964. doi:10.1016/j.jad.2018.11.090 30699881

[200] Wang Y , Wang S , Xin Y , et al. Hydrogen sulfide alleviates the anxiety‐like and depressive‐like behaviors of type 1 diabetic mice via inhibiting inflammation and ferroptosis. Life Sci. 2021;278:119551. doi:10.1016/j.lfs.2021.119551 33945828

[201] Liu HY , Wei HJ , Wu L , et al. BDNF‐TrkB pathway mediates antidepressant‐like roles of H2 S in diabetic rats via promoting hippocampal autophagy. Clin Exp Pharmacol Physiol. 2020;47:302‐312. doi:10.1111/1440-1681.13201 31660632

[202] Jiang W , Tang YY , Zhu WW , et al. PI3K/AKT pathway mediates the antidepressant‐ and anxiolytic‐like roles of hydrogen sulfide in streptozotocin‐induced diabetic rats via promoting hippocampal neurogenesis. Neurotoxicology. 2021;85:201‐208. doi:10.1016/j.neuro.2021.05.016 34087334

[203] Tang ZJ , Zou W , Yuan J , et al. Antidepressant‐like and anxiolytic‐like effects of hydrogen sulfide in streptozotocin‐induced diabetic rats through inhibition of hippocampal oxidative stress. Behav Pharmacol. 2015;26:427‐435. doi:10.1097/FBP.0000000000000143 25932716

[204] Lv S , Liu H , Wang H . Exogenous hydrogen sulfide plays an important role by regulating autophagy in diabetic‐related diseases. Int J Mol Sci. 2021;22:22. doi:10.3390/ijms22136715 PMC8268438 34201520

[205] Chaves YC , Genaro K , Crippa JA , da Cunha JM , Zanoveli JM . Cannabidiol induces antidepressant and anxiolytic‐like effects in experimental type‐1 diabetic animals by multiple sites of action. Metab Brain Dis. 2021;36:639‐652. doi:10.1007/s11011-020-00667-3 33464458

[206] Silote GP , Gatto MC , Eskelund A , Guimaraes FS , Wegener G , Joca SRL . Strain‐, sex‐, and time‐dependent antidepressant‐like effects of Cannabidiol. Pharmaceuticals (Basel). 2021;14:14. doi:10.3390/ph14121269 PMC8709491 34959670

[207] Vong CT , Tseng HHL , Kwan YW , Lee SM , Hoi MPM . Novel protective effect of O‐1602 and abnormal cannabidiol, GPR55 agonists, on ER stress‐induced apoptosis in pancreatic beta‐cells. Biomed Pharmacother. 2019;111:1176‐1186. doi:10.1016/j.biopha.2018.12.126 30841431

[208] Shivavedi N , Charan Tej GNV , Neogi K , Nayak PK . Ascorbic acid therapy: a potential strategy against comorbid depression‐like behavior in streptozotocin‐nicotinamide‐induced diabetic rats. Biomed Pharmacother. 2019;109:351‐359. doi:10.1016/j.biopha.2018.10.070 30399569

[209] Shivavedi N , Kumar M , Tej G , Nayak PK . Metformin and ascorbic acid combination therapy ameliorates type 2 diabetes mellitus and comorbid depression in rats. Brain Res. 2017;1674:1‐9. doi:10.1016/j.brainres.2017.08.019 28827076

[210] Moretti M , Werle I , da Rosa PB , et al. A single coadministration of subeffective doses of ascorbic acid and ketamine reverses the depressive‐like behavior induced by chronic unpredictable stress in mice. Pharmacol Biochem Behav. 2019;187:172800. doi:10.1016/j.pbb.2019.172800 31678791

[211] Mason SA , Rasmussen B , van Loon LJC , Salmon J , Wadley GD . Ascorbic acid supplementation improves postprandial glycaemic control and blood pressure in individuals with type 2 diabetes: findings of a randomized cross‐over trial. Diabetes Obes Metab. 2019;21:674‐682. doi:10.1111/dom.13571 30394006

[212] Boonthongkaew C , Tong‐Un T , Kanpetta Y , Chaungchot N , Leelayuwat C , Leelayuwat N . Vitamin C supplementation improves blood pressure and oxidative stress after acute exercise in patients with poorly controlled type 2 diabetes mellitus: a randomized, placebo‐controlled, cross‐over study. Chin J Physiol. 2021;64:16‐23. doi:10.4103/cjp.cjp_95_20 33642340

[213] Jiang CL , Jen WP , Tsao CY , Chang LC , Chen CH , Lee YC . Glucose transporter 10 modulates adipogenesis via an ascorbic acid‐mediated pathway to protect mice against diet‐induced metabolic dysregulation. PLoS Genet. 2020;16:e1008823. doi:10.1371/journal.pgen.1008823 32453789 PMC7274451

[214] El‐Marasy SA , Abdallah HM , El‐Shenawy SM , El‐Khatib AS , El‐Shabrawy OA , Kenawy SA . Anti‐depressant effect of hesperidin in diabetic rats. Can J Physiol Pharmacol. 2014;92:945‐952. doi:10.1139/cjpp-2014-0281 25358020

[215] Zhu X , Liu H , Liu Y , Chen Y , Liu Y , Yin X . The antidepressant‐like effects of hesperidin in streptozotocin‐induced diabetic rats by activating Nrf2/ARE/glyoxalase 1 pathway. Front Pharmacol. 2020;11:1325. doi:10.3389/fphar.2020.01325 32982741 PMC7485173

[216] Kosari‐Nasab M , Shokouhi G , Ghorbanihaghjo A , Abbasi MM , Salari AA . Hesperidin attenuates depression‐related symptoms in mice with mild traumatic brain injury. Life Sci. 2018;213:198‐205. doi:10.1016/j.lfs.2018.10.040 30352242

[217] Xie L , Gu Z , Liu H , et al. The anti‐depressive effects of hesperidin and the relative mechanisms based on the NLRP3 inflammatory signaling pathway. Front Pharmacol. 2020;11:1251. doi:10.3389/fphar.2020.01251 32922291 PMC7456860

[218] Hanchang W , Khamchan A , Wongmanee N , Seedadee C . Hesperidin ameliorates pancreatic beta‐cell dysfunction and apoptosis in streptozotocin‐induced diabetic rat model. Life Sci. 2019;235:116858. doi:10.1016/j.lfs.2019.116858 31505195

[219] Homayouni F , Haidari F , Hedayati M , Zakerkish M , Ahmadi K . Blood pressure lowering and anti‐inflammatory effects of hesperidin in type 2 diabetes; a randomized double‐blind controlled clinical trial. Phytother Res. 2018;32:1073‐1079. doi:10.1002/ptr.6046 29468764

[220] Bikri S , Aboussaleh Y , Berrani A , et al. Effects of date seeds administration on anxiety and depressive symptoms in streptozotocin‐induced diabetic rats: biochemical and behavioral evidences. J Basic Clin Physiol Pharmacol. 2021;32:1031‐1040. doi:10.1515/jbcpp-2020-0225 33705613

[221] Bai Y , Xin M , Lin J , et al. Banana starch intervention ameliorates diabetes‐induced mood disorders via modulation of the gut microbiota‐brain axis in diabetic rats. Food Agric Immunol. 2022;33:377‐402. doi:10.1080/09540105.2022.2071846

[222] Farimani AR , Hariri M , Azimi‐Nezhad M , Borji A , Zarei S , Hooshmand E . The effect of n‐3 PUFAs on circulating adiponectin and leptin in patients with type 2 diabetes mellitus: a systematic review and meta‐analysis of randomized controlled trials. Acta Diabetol. 2018;55:641‐652. doi:10.1007/s00592-018-1110-6 29453672

